# Extraction, Antioxidant Capacity, 5-Lipoxygenase Inhibition, and Phytochemical Composition of Propolis from Eastern Canada

**DOI:** 10.3390/molecules25102397

**Published:** 2020-05-21

**Authors:** Mariama Sambou, Jacques Jean-François, Fanta J. Ndongou Moutombi, Jérémie A. Doiron, Mathieu P.A. Hébert, Andrew P. Joy, Ngoc-Nu Mai-Thi, David A. Barnett, Marc E. Surette, Luc H. Boudreau, Mohamed Touaibia

**Affiliations:** 1 Department of Chemistry and Biochemistry, Université de Moncton, Moncton, NB E1A 3E9, Canada; mari141@hotmail.fr (M.S.); efn2132@umoncton.ca (F.J.N.M.); jeremie.doiron@umoncton.ca (J.A.D.); mathieu.hebert@umoncton.ca (M.P.A.H.); marc.surette@umoncton.ca (M.E.S.); luc.boudreau@umoncton.ca (L.H.B.); 2 Department of Chemistry, Texas Lutheran University, Seguin, TX 78155, USA; jjean-francois@tlu.edu; 3 Atlantic Cancer Research Institute, Moncton, NB E1C 8X3, Canada; AndrewJ@canceratl.ca (A.P.J.); MaiN@canceratl.ca (N.-N.M.-T.); davidb@canceratl.ca (D.A.B.)

**Keywords:** Eastern Canadian propolis, extraction, total phenolic content, total flavonoids content, phytochemical composition, antioxidant activity, anti-inflammatory

## Abstract

Soxhlet (SE), microwave-assisted (MAE) and ultrasound-assisted (UAE) extraction were compared using ten extraction solvents for their efficiency to extract phenolic and flavonoid antioxidants from Eastern Canada propolis. Extracts were compared for total phenolic (TPC) and total flavonoid (TFC) content, and radical scavenging activities. Anti-inflammatory activity through inhibition of 5-lipoxygenase (5-LO) products biosynthesis in HEK293 cells was also evaluated. The results showed that SE extracts using polar solvents had the highest TPC and TFC. Extracts obtained with ethanol, methanol and acetone were effective free radical scavengers, and showed 5-LO inhibition similar to zileuton. UAE was an effective extraction method since the extracts obtained were comparable to those using SE and the MAE while being done at room temperature. With UAE, extracts of less polar solvents showed similar free radical scavenging and 5-LO inhibition to extracts of much more polar solvents such as methanol or ethanol. Reversed-phase liquid chromatography tandem mass spectrometry confirmed the presence of 21 natural compounds in the propolis extracts based on the comparison of intact mass, chromatographic retention time and fragmentation patterns derived from commercial analytical standards. The current study is the first of its kind to concurrently investigate solvent polarity as well as extraction techniques of propolis.

## 1. Introduction

Propolis is a resinous material produced by bees upon the mixing of their saliva with substances originating from plant sources. The chemical composition of propolis is highly dependent on the geographical location of the harvested plants [1,2], the bee subspecies [3] and the environmental conditions [4] amongst other factors. Propolis is primarily used by bees as a protective sealant to shut off predators and pathogens, to create a sterile environment for their larvae and to isolate the beehive against unfavorable weather conditions [5]. Irrespective of origin, the propolis mixture usually contains waxes, resins, aromatic and ethereal oils and other organic substances [6] making it a highly hydrophobic substance that explains its use as a sealant by bees. As for many natural products, although not viewed as a conventional drug because of its lack of standardization, it has long ago found a niche in traditional and folk medicine because of its many beneficial attributes including antibacterial and anti-inflammatory properties amongst others. These properties, in turn, have led to the use of propolis in several therapeutic strategies such as anticancer [7], antibacterial [8,9], antiviral [10], and anti-inflammatory [11]. Other current leads for therapeutic strategies using propolis include applications as diverse as a periodontal biomaterial for restorative oral surgery [12] or wound healing in ulcerative diabetic wound treatment [13]. Most of those properties stem from the fact that a majority of the aforementioned pathologies have an oxidative nature characterized by the overproduction of reactive oxygen species (ROS) such as hydrogen peroxide, superoxide anion, hydroxyl ions and nitric oxide. The subsequent results of this oxidative nature are deleterious effects on cell biomolecules such as lipids, proteins and nucleic acids leading to cell modification and death. In propolis, two groups of compounds, flavonoids and phenolic acids [14], are at the basis of the observed antioxidant properties and by extension of its beneficial therapeutic properties. These 2 groups of antioxidant molecules are characterized by their ability to scavenge free radicals using the phenol groups present in their structure counteracting the oxidative stress common to many pathologies. The flavonoids are derivates with a polyphenolic structure with various subclasses whereas, the phenolic acids are characterized by the presence of both a phenol and a carboxylic acid group. Other non-phenolic substances found in propolis that exhibit an antioxidant activity include the amyrins with a triterpenoid structure [15].

The success of propolis as a dietary supplement has led to an increased interest in its chemical composition. The chemical composition is extremely dependent on the plants found around the hive, as well as on the geographic and climatic characteristics of the collection site.

The objectives of the current study are as follow. First, to evaluate the effect of different extraction methods and solvents on the type of extracted phytochemicals as well as on the quantities obtained from propolis native to the Canadian province of New Brunswick. 

In a second phase, to characterize the total phenolic content (TPC) and total flavonoid content (TFC) of extracts of this propolis that has not been previously characterized. In a third phase, to evaluate the anti-inflammatory properties of extracts from this propolis by performing inhibition studies of the enzyme 5-lipoxygenase (5-LO). 5-LO is a validated target for the treatment of inflammation and related disorders [16]. Given the weak potency, hepatotoxicity and unfavourable pharmacokinetic profile of zileuton (Zyflo^®^), currently the only approved and clinically used 5-LO inhibitor [17], the search for potent and safe 5-LO inhibitors is highly demanded. Recent studies from our group have shown that caffeic acid phenethyl ester (CAPE), a component of propolis, is significantly more potent than zileuton [18]. 

Finally, use of reversed-phase liquid chromatography coupled to high-resolution/accurate mass (HR/AM) mass spectrometry to characterize and identify the major chemical components of propolis extracts from this geographic region.

## 2. Results and Discussion

### 2.1. Extractions

For comparison, we used the following three extraction methods: soxhlet extraction (SE), microwave-assisted extraction (MAE), and ultrasonic-assisted extraction (UAE) were tested. As the solvent’s polarity has a considerable influence on the type of the extracted phytochemicals, ten different solvents were used for extraction. For a statistical analysis, each extraction was done in triplicate. The ten solvents/mixtures are: water, methanol, ethanol, acetone, ethyl acetate (EtOAc), dichloromethane (DCM), ethyl acetate-hexane (1:1), ethyl acetate-hexane (4:1), ethyl acetate-hexane (1:4) and hexane. All solvents were compared using the three extraction methods.

Most of the substances found in propolis are lipophilic and unsurprisingly the best extraction yields for propolis, irrespective of the extraction method used, were obtained in polar organic solvents such as ethanol, methanol, acetone, ethyl acetate and dichloromethane (Table 1). 

Usually, polar solvents favor better extraction of propolis and by extension improve the antioxidant properties of extracts when compared to non-polar solvents [19]. Extraction yield decreased in the presence of a non-polar organic solvent such as hexane and was minimal in the presence of water. Amongst the three methods used in this study for propolis extraction, the SE method showed consistently better extraction yield irrespective of the type of solvent used when compared to the two other extraction methods. The slightly higher yield for the SE method might be explained by a higher extraction time, 6 h vs. 10 min for the other two methods. 

### 2.2. Total Phenolic Content

The sought-after anti-oxidative properties of propolis rely on the presence of polyphenols, a specific class of phytochemicals which encompass a large group of metabolites found in fruits, vegetables and propolis [20,21]. The most abundant polyphenols usually present in propolis are flavonoids and phenolic acids. Flavonoids are tricyclic structures consisting of two phenolic rings and a pyran moiety, whereas phenolic acids, although presenting a wide variety of structures, usually have an aromatic ring and a carboxyl group [14].

**Figure 1:**
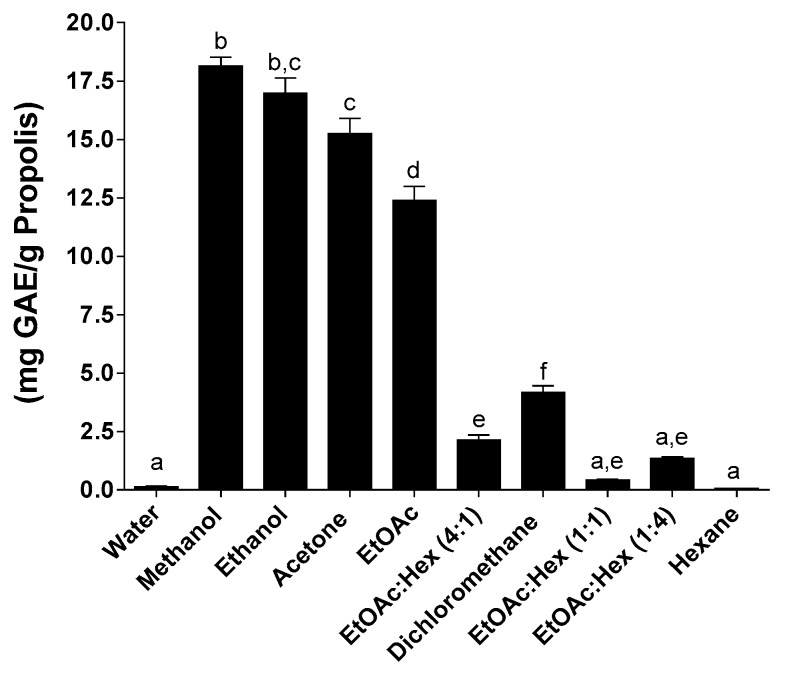
Total phenolic content following SE. Values are the means ± SEM of at least three independent experiments, each performed in duplicate; values with different superscripts are significantly different (*p* < 0.05) as determined by one-way ANOVA test with subsequent Tukey’s adjustment; GAE: gallic acid equivalent.

**Figure 2:**
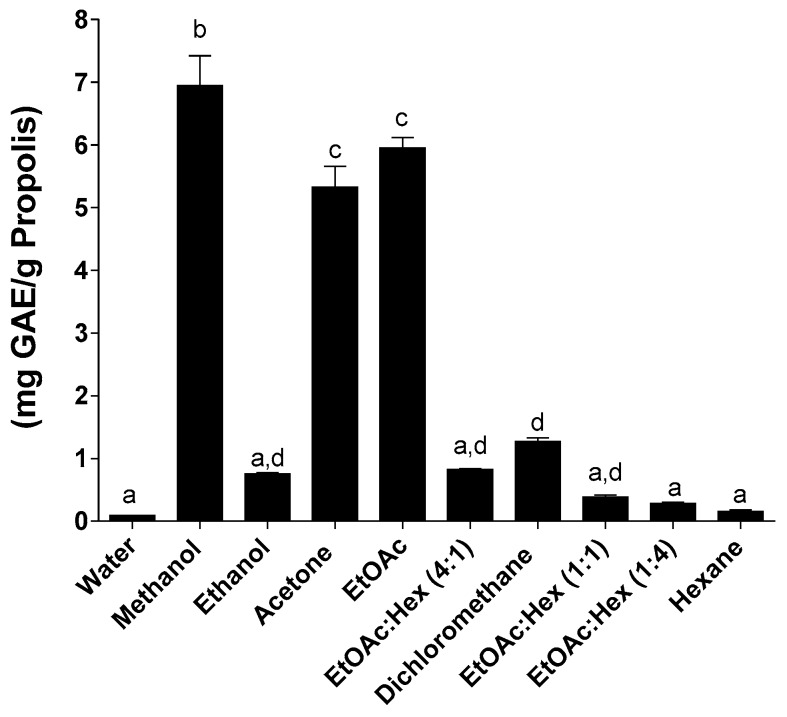
Total phenolic content following MAE. Values are the means ± SEM of at least three independent experiments, each performed in duplicate; values with different superscripts are significantly different (*p* < 0.05) as determined by one-way ANOVA test with subsequent Tukey’s adjustment; GAE: gallic acid equivalent.

**Figure 3:**
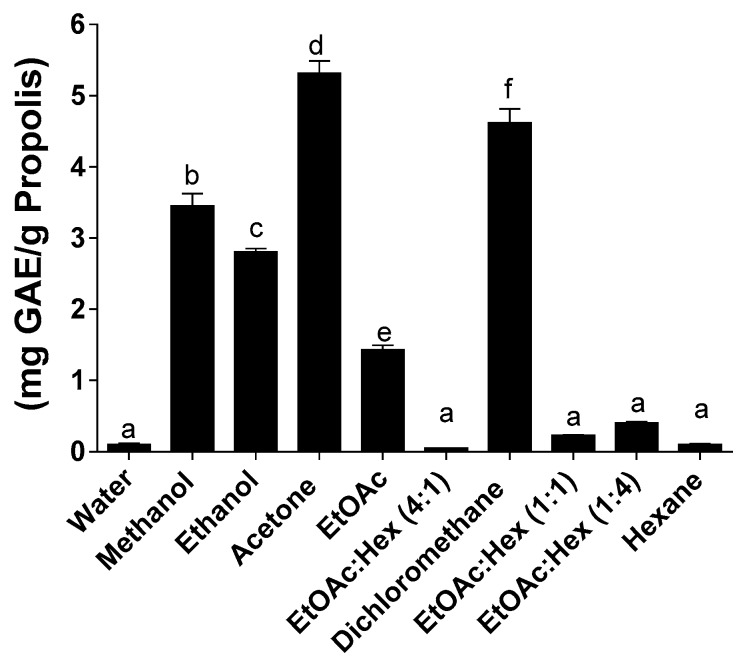
Total phenolic content following UAE. Values are the means ± SEM of at least three independent experiments, each performed in duplicate; values with different superscripts are significantly different (*p* < 0.05) as determined by one-way ANOVA test with subsequent Tukey’s adjustment; GAE: gallic acid equivalent.

Total phenolic content mirrored propolis yields when comparing the three extraction methods used in this study (Figure 1 Figure 2 Figure 3). Partition of phenolic compounds in methanol or ethanol using the SE method was at least twice the observed partition of phenolic compounds in the same solvents when using the MAE or UAE methods. 

In ethanol the MAE yielded significantly lower TPC content (GAE: gallic acid equivalent/g propolis) when compared to SE and UAE. The same pattern was repeated in dichloromethane with statistically significant lower TPC content compared to the two other methods. These results are in line with current data from the literature where the most used solvent in propolis extraction is ethanol. The ethanolic extracts of propolis tend to have a high content in polyphenolic compounds and flavonoids [22,23] and low contents in waxes and other side products normally found in propolis [24]. Quantification of total phenolic content for SE revealed that methanol, ethanol, acetone and ethyl acetate, polar organic solvents, were the best solvents since they outperformed all other solvents and solvent mixtures (Figure 1). Hexanes, alone or containing 25% or 50% ethyl acetate, and water were the least efficient solvents or mixtures for the extraction of phenolic compounds. Dichloromethane, as well as ethyl acetate containing 25% hexane, allowed moderate extraction of polyphenols (Figure 1). 

The use of the MAE or UAE methods lead to a drastic reduction, by at least half, of the phenolic content compared to SE as shown in Figure 2 and Figure 3. We also report statistically significant low total phenolic content for ethanol and dichloromethane when using the MAE method. Since this decrease of the phenolic compounds for MAE and UAE is not matched by an equivalent decrease in the extraction yield of propolis, other factors might come into play to explain these results. Many of the propolis components contain aromatic rings or double bonds that might be susceptible to the energy provided by the microwave-assisted extraction method. This is in line with the results reported earlier by Trusheva et al. [25].

Highlighting the differences in composition based on geographical location, the best values of TPC (15–20 mg GAE/g propolis) of our propolis originating from apiaries in Eastern Canada is much lower than the TPC of red propolis (333 mg GAE/g propolis) harvested from apiaries located in Northeastern Brazil when ethanol is used for extraction [26]. All three methods in this study used, at some stage, some amount of heat or energy that might be detrimental to the activity of the biologically active compounds found in propolis. So new non–thermal and greener methods are actively being developed to increase yield in an environmentally-friendly manner [24,27]. At this stage a good correlation seems to exist between the extraction method and the TPC, the most efficient extraction method, the SE, also yields the highest TPC contents for at least four out of the five most efficient solvents used.

### 2.3. Total Flavonoid Content

**Figure 4:**
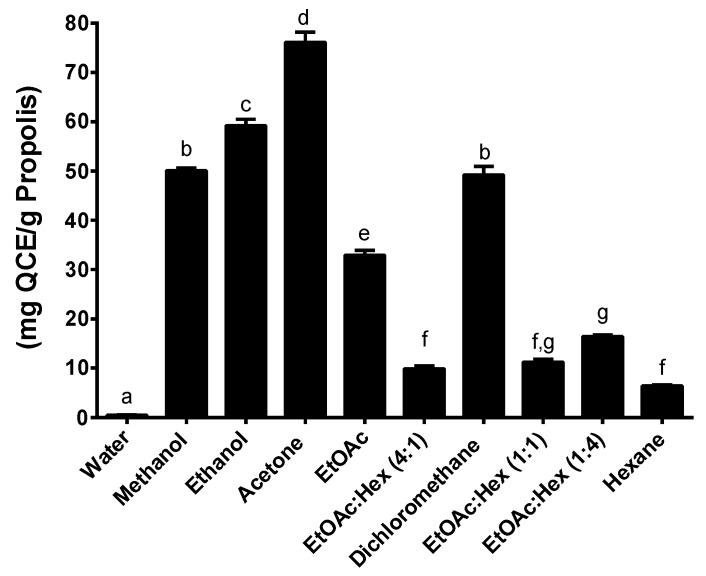
Total flavonoid content following SE. Values are the means ± SEM of at least three independent experiments, each performed in duplicate; values with different superscripts are significantly different (*p* < 0.05) as determined by one-way ANOVA test with subsequent Tukey’s adjustment; QCE: quercetin equivalent.

**Figure 5:**
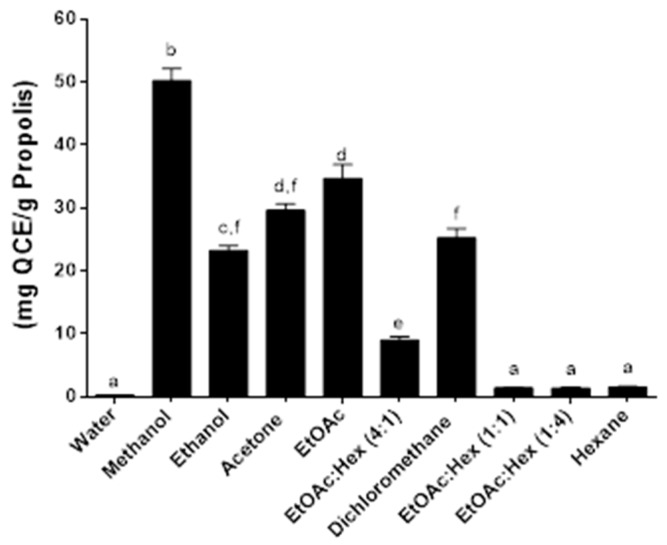
Total flavonoid content following MAE. Values are the means ± SEM of at least three independent experiments, each performed in duplicate; values with different superscripts are significantly different (*p* < 0.05) as determined by one-way ANOVA test with subsequent Tukey’s adjustment; QCE: quercetin equivalent.

**Figure 6:**
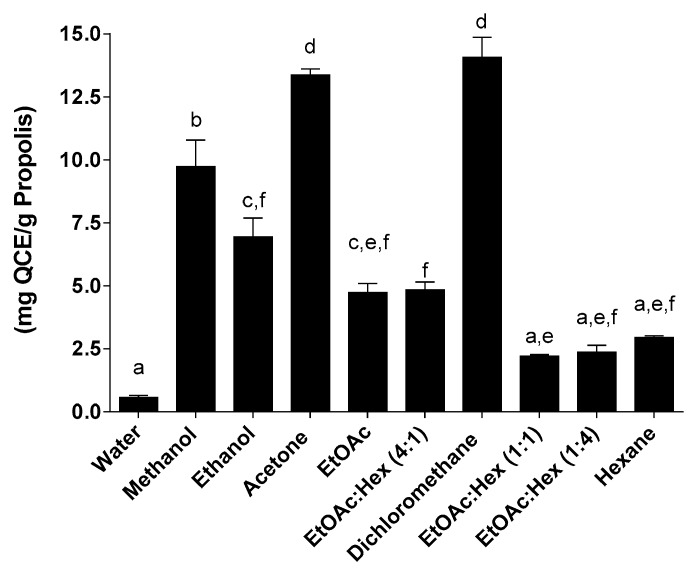
Total flavonoid content following UAE. Values are the means ± SEM of at least three independent experiments, each performed in duplicate; values with different superscripts are significantly different (*p* < 0.05) as determined by one-way ANOVA test with subsequent Tukey’s adjustment; QCE: quercetin equivalent.

The flavonoid quercetin was used for the determination of total flavonoid content of the propolis extracts and total flavonoid content was expressed as quercetin equivalents (mgQCE/g propolis) (Figure 4, Figure 5 and Figure 6). In a pattern similar to the total phenolic content, the total flavonoid content yields the best results when using the SE method (Figure 4); higher TFCs were generated with the SE, as well as with MAE, essentially with the more polar solvents. Of the three extraction methods used, the UAE has always had a lower TFC compared to the other two methods. The TFC of our extracted propolis (Eastern Canada apiaries), factoring in the different extraction methods using ethanol, ranged from 10 to 60 mg QCE/g and compares well with the TFC of different Brazilian propolis such as red propolis (187 mg QCE/g) or brown propolis (30 mg QCE/g) [28]. Overall the best yields of propolis obtained by the SE method are mirrored by the highest TPC and TFC amongst the 3 extraction methods used in this study. A certain pattern seems to emerge where high propolis yields correlate with both high TPC and TFC at least for the SE extraction method. The search for greener and faster alternative methods for propolis extraction has led to the development, amongst others, of ultrasound-assisted extraction and supercritical fluid extraction with carbon dioxide. Ultrasound is widely recognized as a reliable and fast method to extract various compounds from their natural matrices but has only very recently been applied to propolis extraction [29,30]. 

Results from our study have shown a consistent drop in TPC and TFC when comparing UAE with SE. This contradicts a recent study by Reis et al. [26] that shows similar or higher TPC and TFC when comparing UAE with a conventional method (ethanol extraction). 

The disparity might be explained in terms of the experimental parameters: our study used twice the amount of propolis i.e., 5 g against 2 g in the Reis study for roughly similar volumes of ethanol. Perhaps more importantly, the ultrasound was applied for two periods of 10 min in our study against 50 min at 50 °C for the Reis study [26].

### 2.4. Antioxidant Activity

Antioxidant properties are one of the most sought-after characteristics of natural products such as propolis. The antioxidant activity of the various propolis extracts was expressed in terms of their free radical scavenging activity as measured by the 2,2-diphenyl-1-picrylhydrazyl (DPPH•) radical method (Table 2). 

Low IC_50_ values reflect high antioxidant activity and three known antioxidants ascorbic acid, caffeic acid and quercetin were used as reference compounds. Mirroring previous results for propolis yields (TPC and TFC in the presence of water and hexane), antioxidant activity was low in the presence of water or could not be determined in hexane (very high IC_50_ values) whatever the extraction method used. Extracts obtained with polar organic solvents such as methanol and ethanol showed the highest antioxidant activity of all the solvents used, independently of the extraction method. For the methanolic or ethanolic extracts, IC_50_ values hover between 59–79 µg/mL, around 7-10 times the IC_50_ value of caffeic acid. Acetone extracts obtained with an UAE showed the highest antioxidant potential (IC_50_ = 48 µg/mL). The effect of ultrasound, even at room temperature, seems to favor the extraction of molecules with high antioxidant activity. Considering that propolis extracts are still complex mixtures of different compounds, it is difficult to pinpoint the most effective ingredient and one must consider the fact that all the ingredients in a propolis extract could act synergistically in vivo.

If we compare the best antioxidant activities, in the presence of ethanol or methanol, versus the extraction method used, there is no clear pattern and for a particular solvent the IC_50_ values for the 3 extraction methods lay in a similar range or overlap. In a surprising reverse, UAE which showed low propolis extraction yields and consistently low TPC and TFC seems at least for 2 solvents (acetone and ethyl acetate) to be the best approach to generate propolis extracts with potent antioxidant activity. Our antioxidant activities in this sense are similar to recent results reported by Reis et al. [26] who showed similar antioxidant activities using either a conventional ethanolic method or an ultrasound-assisted method. Our results differ from Devequi-Nunes who showed consistently higher antioxidant activity for a fluid supercritical extraction method than a conventional ethanolic extraction method [29]. This difference highlights the importance of the extraction method on parameters such as TPC, TFC and the antioxidant activity.

### 2.5. 5-LO Product Biosynthesis Assays in HEK293 Cells

5-LO is an iron-dependent enzyme found in all blood leukocytes and it has an important role in cell-based inflammatory responses making it a key target in inflammation-based disease pathways [31]. 

**Figure 7:**
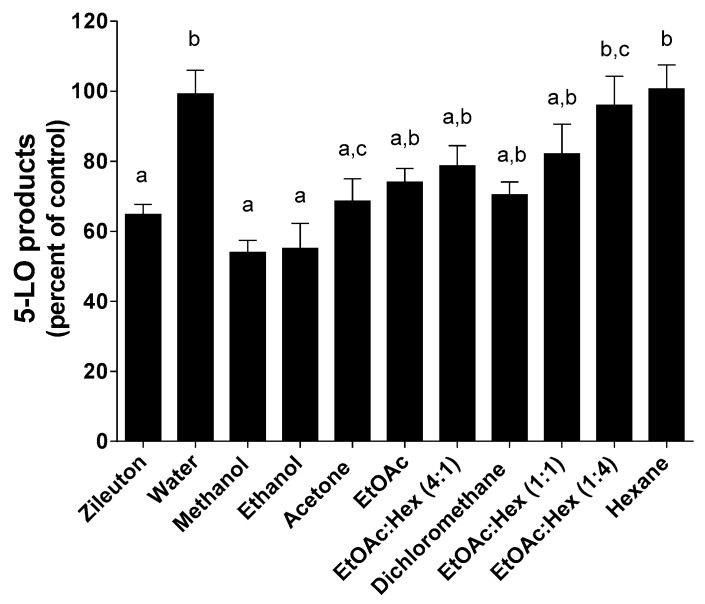
Inhibition of 5-LO product biosynthesis in HEK293 cells by Zileuton (1 µM) and all dry extracts (5 µg/mL) obtained following a SE. Values are the means ± SEM of at least three independent experiments, each performed in duplicate; values with different superscripts are significantly different (*p* < 0.05) as determined by one-way ANOVA test with subsequent Tukey’s adjustment.

**Figure 8:**
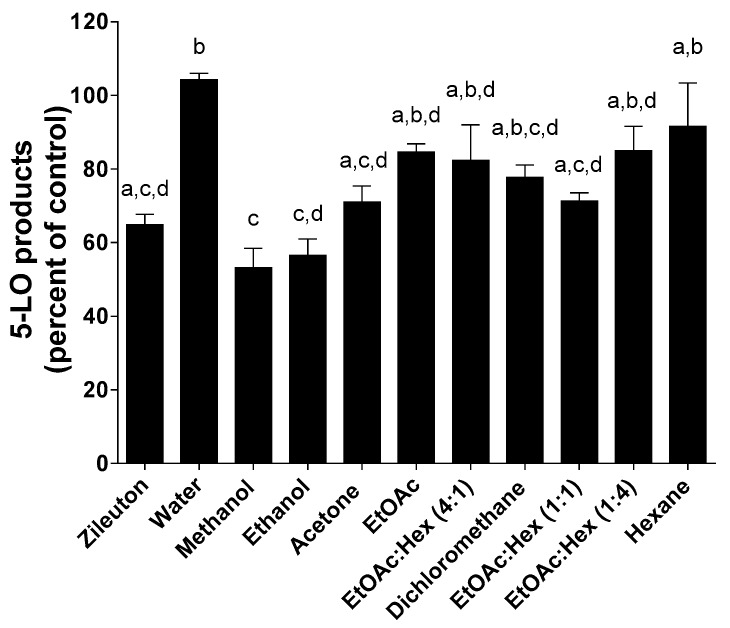
Inhibition of 5-LO product biosynthesis in HEK293 cells by zileuton (1 µM) and all dry extracts (5 µg/mL) following MAE. Values are the means ± SEM of at least three independent experiments, each performed in duplicate; values with different superscripts are significantly different (*p* < 0.05) as determined by one-way ANOVA test with subsequent Tukey’s adjustment.

**Figure 9:**
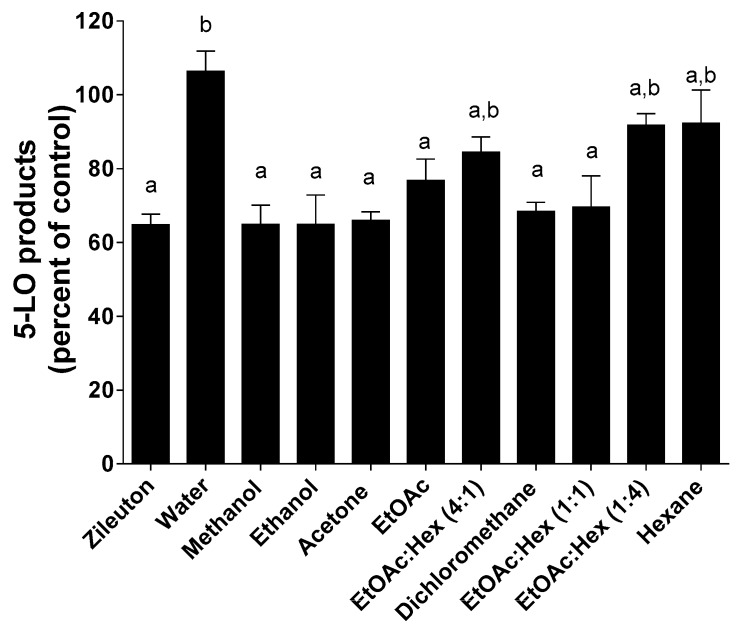
Inhibition of 5-LO product biosynthesis in HEK293 cells by zileuton (1 µM) and all dry extracts (5 µg/mL) following UAE. Values are the means ± SEM of at least three independent experiments, each performed in duplicate; values with different superscripts are significantly different (*p* < 0.05) as determined by one-way ANOVA test with subsequent Tukey’s adjustment.

Because of 5-LO pathway involvement in a variety of human diseases, the design of 5-LO inhibitors is an active field but to date, only one FDA-approved 5-LO inhibitor, zileuton, is clinically available. Because of their known antioxidative properties, propolis extracts and pure compounds from propolis have been investigated as potential inhibitors of 5-LO. HEK293 cells stably transfected with both 5-LO and 5-LO-activating protein (FLAP) were used to screen propolis extracts for 5-LO inhibition (Figure 7, Figure 8 and Figure 9) as previously described [32]. 

As shown in Figure 7, methanol, ethanol and acetone SE extracts were the only ones that showed a significant decrease in 5-LO product biosynthesis compared to controls. A concentration of 5 µg/mL of these extracts was equipotent to 1 µM zileuton. All remaining extracts prepared with less polar solvents as well as hexane/ethyl acetate mixtures were equipotent to less active than 1 µM zileuton. As expected, water extracts show no activity on the inhibition of 5-LO product biosynthesis in HEK293 cells. Except for water, solvent polarity following a SE appears to be a determining factor for the extraction of molecules with 5-LO inhibitory activity.

As shown in Figure 8, the effect of extracts on 5-LO product biosynthesis obtained following a MAE appears to be similar to those of a SE. 

The gain in time of extraction can be considerable since a MAE takes only minutes compared to a SE that can take hours. The polarity of the solvents is also a determining factor as to the anti-5-LO activity of the extracts obtained following MAE. Surprisingly, extracts obtained with ethyl acetate/hexane (1:1) mixture is equipotent with that obtained with acetone (Figure 8). These two extracts have the same activity as extracts obtained with ethanol and methanol.

As with the other two extraction methods (SE and MAE), extracts obtained following UAE with the three most polar organic solvents show good 5-LO products biosynthesis inhibitory activity and are equipotent with 1 µM zileuton (Figure 9). Contrary to extracts obtained with SE and MAE, extracts obtained following UAE with ethyl acetate and dichloromethane significantly inhibit the biosynthesis of 5-LO products compared to control. The effect of ultrasound, even at room temperature, seems to favour the extraction of molecules with anti 5-LO activity even with less polar solvents than alcohols.

At this stage when trying to correlate propolis extraction yield with other parameters, it appears, at least for the SE method, that there is a direct correlation between propolis yield and TFC and TPC. But when it came down to antioxidant activity and 5-LO anti-inhibitory activity that pattern broke down and all 3 methods seem to be more or less equivalent. Although the SE method is able to yield a higher amount of flavonoids and phenolics, the polyphenols that mattered for antioxidant activity and anti-5-LO activity, are apparently extracted with the same efficiency independently of the extraction method used.

Inhibition of 5-LO product biosynthesis is not directly related to the cytotoxicity against HEK293 cells since the extracts obtained with highly polar solvents exhibited no cytotoxic effect (see Figure S1 in the Supplementary Material).

### 2.6. Identification of Chemical Compounds in Propolis from Eastern Canada Obtained Following SE, MAE and UAE

Chemical profiles of propolis from Eastern Canada obtained from three extraction methods were examined using microscale liquid chromatography interfaced to a high-resolution/accurate mass quadrupole-Orbitrap mass spectrometer. Phenolic acids and flavonoids are readily observed by mass spectrometry as their pseudo-molecular anions. All mass spectral data were therefore acquired in negative ion mode at a maximum resolution setting of 140,000 (@ *m*/*z* 200). The gradient LC method was initially optimized for seven compounds suspected to be present in propolis, namely, caffeic acid, gallic acid, *p*-coumaric acid, ferulic acid, cinnamic acid, quercetin, and caffeic acid phenyl ester (CAPE). While gallic acid was also used for the total phenolic acid assay, it was not actually found to be present in any of the propolis extracts. Compared to the other six standards, gallic acid was also more hydrophilic and poorly retained on the C-18 micro-column. Following the initial LC-MS/MS method development with the seven standards, an ultrasound-assisted ethanol extract was tested to tentatively identify other precursor ions from other abundant compounds. Based on this analysis and a comparison to several other published lists of propolis components [33,34,35,36,37,38,39], seventeen more standards were purchased from Sigma-Aldrich (Mississauga, ON, Canada). All twenty-four standards (including gallic acid) were tested by LC-MS/MS on the quadrupole-Orbitrap and by infusion on a linear quadrupole ion trap mass spectrometer (LTQ-XL, Thermo-Fisher Scientific, San Jose, CA, USA). The characteristic information used to confirm the presence of twenty-one of these compounds in propolis is listed in Table 3. 

Table 3 gives all of the chemicals by their common name; CAS number; retention time; measured mass; calculated mass accuracy (ppm) and three most abundant fragment ions. Characteristic fragments were determined by HCD (high-energy collision-induced dissociation) on the quadrupole-Orbitrap and by CID (collision-induced dissociation) on the linear quadrupole ion trap. Note that 3,4-dimethoxycinnamic acid was absent from all extracts and thus omitted from Table 3. The standard compound pinostrobin was also found to be insoluble in ethanol. Dissolution of pinostrobin in dimethylsulfoxide (DMSO) permitted its analysis by LC-MS but no evidence of this compound was found in any of the ethanol soluble propolis extracts. 

In addition to these 21 confirmed compounds, we also used exact mass information collected at high resolution to predict the empirical formulae of an additional 25 high abundance (i.e., >9 × 10^6^) compounds that were common to almost every extract.

A list of these abundant, but unidentified, compounds is given in Table 4 in the order that they elute from the LC column. Table 4 includes the observed mass, the predicted empirical formula, the mass accuracy between the observed and calculated mass and the three most abundant fragments. This list of ions was compared to additional lists of propolis composition [40,41,42] but the only tentative assignment was for *m*/*z* 177.0533 at a retention time of 16.3 min identified as 3-methoxy-4-hydroxycinnamaldehyde. Note that the mass accuracy was within 5 ppm for all but 4 of the predicted empirical formulae. 

### 2.7. Semi-Quantitative Analysis of 46 Compounds in Propolis by HPLC-MS Analysis

A total of 24 extracted samples of propolis representing three different extraction methods and eight unique solvents were analyzed by LC-MS. Raw signals were recorded for the 46 analytes listed in Table 3 and Table 4. The identities of 21 of these compounds were confirmed with analytical standards while empirical formulae were predicted for an additional 25 compounds based on exact mass measurements. The data are described as semi-quantitative in the sense that we did not (a) establish external calibration curves, (b) implement internal standards or (c) evaluate the linearity of response for any of the compounds. Thus, we do not attempt to infer relative abundance between extracted compounds in one sample; rather we choose to illustrate comparisons of individual compounds between samples. 

**Figure 10:**
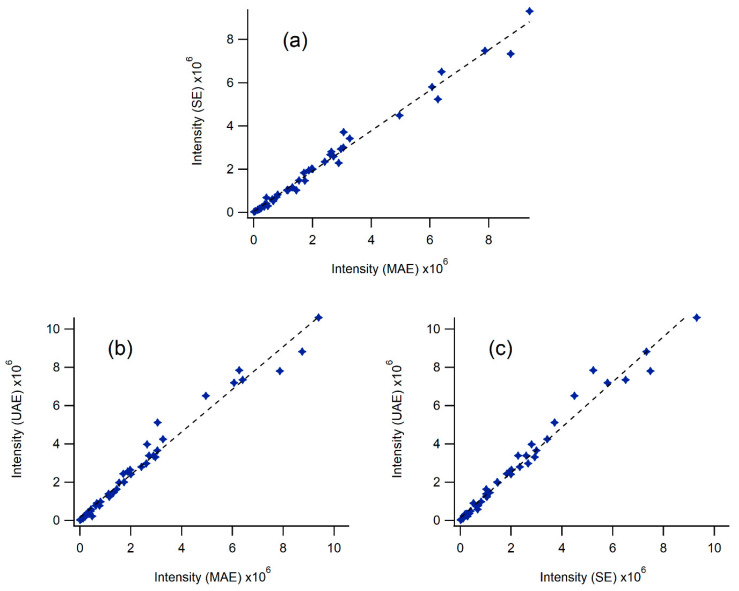
Comparison of LC-MS signals for 45 compounds using SE, UAE and MAE extraction techniques with acetone as the solvent. The identities of twenty of these compounds were confirmed with analytical standards. The signal for p-coumaric acid was omitted from these graphs because of its high abundance. The comparisons are as follows: (**a**) SE *versus* MAE, (**b**) UAE *versus* MAE and (**c**) UAE *versus* SE.

Figure 10 shows three plots comparing (a) SE to MAE, (b) UAE to MAE and (c) UAE to SE for 45 compounds extracted in acetone. The x- and *y*-axis have been normalized to the same value to show the near-equivalency of the three methods with the slopes of (a), (b), and (c) being 0.94, 1.12 and 1.18, respectively. The scatter of the data is also rather low with R^2^ values to the fit of the three plots of 0.985, 0.974 and 0.977. Figures S2–S8 (Supplementary Material) provide simple visual representations of extraction efficiency for the other seven solvents. Steep curves favor the extraction technique plotted on the ordinate while shallow curves favor the abscissa.

**Figure 11:**
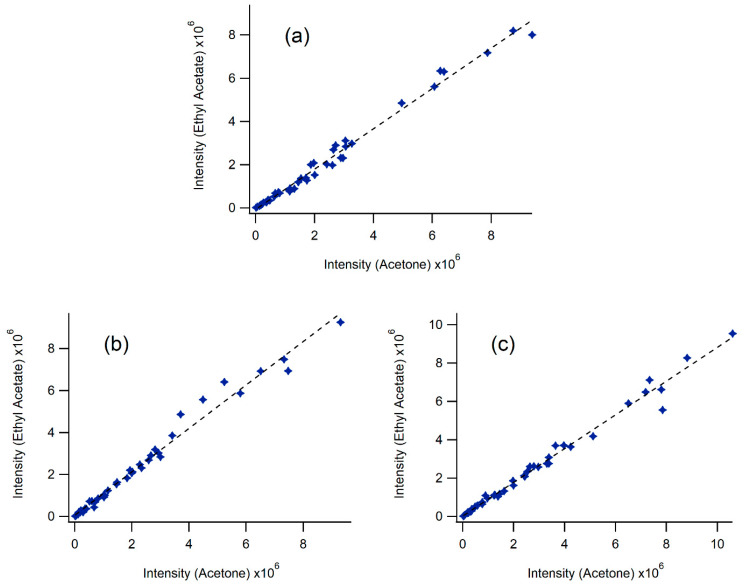
A comparison of LC-MS signals from samples prepared using ethyl acetate and acetone with (**a**) microwave-assisted, (**b**) Soxhlet and (**c**) ultrasound-assisted extraction techniques.

Figure 11 depicts analyte abundance comparisons between ethyl acetate and acetone by the three extraction methods. 

The results are very similar with slopes of 0.93, 1.04 and 0.88 and R^2^ values of 0.988, 0.983 and 0.986, respectively. Tables S1–S3 (Supplementary material) provide summaries of the slope and R^2^ values between solvents for each of the three extraction techniques. The best correlations in terms of unity slopes with minimal scatter of the data (R^2^ > 0.9) tend to be for the more polar solvents methanol, ethanol, acetone and ethyl acetate. 

## 3. Materials and Methods

### 3.1. Materials

Propolis was harvested in Irishtown (Westmorland County, NB, Canada) (46°16′35.04″N, 66°42′50.76″W, 172 m altitude), between September and October 2016 by Acadien Apiaries Ltd. Solvents (methanol, ethanol, acetone, ethyl acetate, hexane) used in the extractions were purchased from VWR Canada (Mississauga, ON, Canada). Folin-Ciocalteu reagent, 2,2-bipyridyl, 2,2-diphenyl-1-picrylhydrazyl radical (DPPH•), quercetin, gallic acid, and calcium ionophore A23187, standards used for LC-MS (see Table 3) were purchased from Sigma-Aldrich (Oakville, ON, Canada). Hank’s balanced salt solution (HBSS) and arachidonic acid were purchased from Lonza (Walkerville, MD, USA) and Cayman Chemical (Ann Arbor, MI, USA), respectively.

### 3.2. Soxhlet Extraction (SE)

Water, methanol, ethanol, acetone, ethyl acetate, dichloromethane, ethyl acetate-hexane (1:1), ethyl acetate-hexane (4:1), ethyl acetate-hexane (1:4) and hexane were used and compared in Soxhlet extraction. The propolis samples (5 g) were placed in a thimble and extracted with each solvent (200 mL) in a Soxhlet extractor for 6 h at solvent reflux temperature. To remove waxes and less soluble substances, suspensions were frozen at −20 °C for 24 h, and then filtered. Solutions were evaporated to near dryness on a rotary evaporator under reduced pressure at room temperature and dried under vacuum (0.1–0.2 mmHg, 24–48 h until reaching constant weight).

### 3.3. Ultrasound-Assisted Extraction (UAE) 

The propolis samples (5 g) were placed in a tube containing the same ten solvents (20 mL) used in SE and extracted each time with an ultrasound liquid processor (Sonicator: Qsonica ultrasound processor, Qsonica L.L.C, Newtown, CT, USA, equipped with a probe). The power delivered into the extraction system was 40 W (at 20% amplitude) for 10 min. The tip of the ultrasound probe was inserted at two thirds of the height of the extraction solvent. To remove waxes and less soluble substances, suspensions were frozen at −20 °C for 24 h and then filtered. Solutions were evaporated to near dryness on a rotary evaporator under reduced pressure at room temperature and dried under vacuum (0.1–0.2 mmHg, 24–48 h until reaching constant weight).

### 3.4. Microwave-Assisted Extraction (MAE) 

The microwave extraction consisted of mixing 5 g of propolis with 20 mL of the same ten solvents used in SE and extracted each time in a flask at a frequency of 70 watts in a domestic microwave oven for 10 min (5 min, twice). The mixture was then filtered and processed like the other procedures. 

### 3.5. Total Phenolic Content (TPC)

Gallic acid was used to construct a standard curve. Different dilutions of 0.25, 0.5, 1, 2, 3 mg/mL were prepared in methanol using gallic acid. One hundred µL of every dilution was mixed with 500 µL of water and then 100 µL of Folin-Ciocalteu. Six minutes later, 1 mL of a solution of sodium carbonate (7%) was added, then 500 µL of water. The solutions were placed in the dark for 90 min. After that, 100 µL of every dilution was plated in triplicate in a 96 well plate and the absorbance was then measured with a multiplate spectrophotometer (VarioScan spectrophotometer, Thermo-Fisher Scientific, San Jose, CA, USA) at 760 nm. The same procedure was used for the determination of total phenolic content in propolis extracts. In this case, 1 mg of extract was diluted in 1 mL of methanol then 100 µL of the solution was used for the assay. The total phenolic content of the propolis was calculated as gallic acid equivalents (mgGAE/g).

### 3.6. Total Flavonoid Content (TFC)

Quercetin was used for the determination of total flavonoid content. A standard curve was determined by preparing different dilutions of 0.5, 1, 5, 7 and 10 mg/mL of quercetin in methanol. One hundred µL of each dilution was mixed with 500 µL of distilled water then with 100 µL of a solution of 5% sodium nitrate. Six minutes later, 150 µL of 10% aluminum chloride solution was added and 5 min later, 200 µL of a solution of 1 M sodium hydroxide. One hundred µL of every dilution was plated in triplicate in a 96 well plate and the absorbance of the solution was measured with a multiplate spectrophotometer at 510 nm. The same procedure was used for the determination of total flavonoid extract in propolis extracts. In this case, 1 mg of extract was diluted in 1 mL of methanol and 100 µL of the solution was used for the assay. The total flavonoid content was determined as quercetin equivalents (mgQCE/g).

### 3.7. Scavenging of Free Radicals

Different concentrations of dried propolis extracts (1, 5, 10, 25, 50, 100 and 125 µg/mL) were diluted in 95% ethanol. The mixture was prepared by mixing 200 µL of ethanol DPPH solution (250 µM) with 200 µL of the fractions of propolis. The tubes were shaken vigorously, held in the dark for 30 min at room temperature and the absorbances were measured at 517 nm using a multiplate spectrophotometer. The DPPH was used as A control, the ethanol as a blank, ascorbic acid, caffeic acid and quercetin were used as positive controls. The radical scavenging activity of the fractions was expressed as a percent and was calculated using the formula:

% Inhibition = [(Acontrol − Atest)/Acontrol] × 100

### 3.8. 5-LO Product Biosynthesis Assays in HEK293 Cells

HEK293 cells were stably co-transfected with a pcDNA3.1 vector expressing 5-LO and a pBUDCE4.1 vector expressing 5-LO-activating protein (FLAP) as previously reported [32,43]. The resulting stable double transfectants were propagated in culture and aliquots were frozen. Once thawed for a series of experiments, each aliquot of cells is cultured for a maximum of 6 weeks before being discarded. For cell stimulation of 5-LO products, transfected HEK293 cells were collected following trypsinization, washed, and the cell pellet was re-suspended in Hank’s balanced salt solution (HBSS) (Lonza) containing 1.6 mM CaCl_2_ at a concentration of 10^6^ cells/mL. Dry propolis extract (5 mg) was diluted in 1 mL of DMSO and 1 µL of this solution was transferred to a 5 mL polypropylene tube. A volume of 1 mL of cell suspension prepared in HBSS/CaCl_2_ (1.6 mM) was then added, gently mixed with a vortex and incubated at 37 °C for 5 min. Cells were then stimulated for 15 min at 37 °C with the addition of 10 μM calcium ionophore A23187 (Sigma-Aldrich) and 10 μM arachidonic acid (Cayman Chemical). The reaction was stopped by adding 0.5 volume of cold stop solution (MeOH:MeCN, 1:1), containing 200 ng/mL of 19-OH prostaglandin B_2_ (PGB_2_) as internal standard. Samples were vortexed and frozen (−80 °C) to maximize protein denaturation, until reversed-phased high-performance liquid chromatography (RP-HPLC) processing as described previously [32]. Data are expressed as means ± standard error of the mean (SEM) of three independent experiments, each performed in duplicate.

### 3.9. Identification of Chemical Profiles by LC-MS/MS Analysis

The chemical profiles of several propolis extracts were analyzed by gradient microscale liquid chromatography (Ultimate 3000, Thermo-Fisher Scientific, San Jose, CA, USA) coupled to a high-resolution/accurate mass quadrupole-Orbitrap mass spectrometer using a pneumatically-assisted electrospray ionization ion source. Electrospray ionization has previously been used to “fingerprint” propolis by mass spectrometry using negative ion mode [34]. Complex extract samples were efficiently fractionated using on-line gradient liquid chromatography with a 10 cm long, 1.0 mm diameter reversed-phase (Altima C18) column with 3 µm particles (VWR, Mississauga, ON, Canada). Solvents “A” and “B” were delivered to the column at a combined flow rate of 40 µL/min. Solvent “A” consisted of 0.1% aqueous formic acid while solvent “B” was composed of 90/9.9/0.1 v/v/v acetontrile/water/formic acid. The LC gradient began at 5%B and was ramped to 100%B over 20 min. The column was then flushed at 100% B for 5 min and re-equilibrated at 5%B for an additional 15 min prior to the injection of the next sample. Each extract was first dissolved in 500 µL ethanol and then diluted 20-fold in 50/50 ethanol/water. Note that attempts to dilute the extracts in a more aqueous buffer for better sample stacking on the reversed-phase column resulted in precipitation. For compound detection, the quadrupole-Orbitrap mass spectrometer was operated in negative ion mode at the maximum resolution setting of 140,000 over a mass-to-charge range of 100–700 Thomson (Th). The mass spectrometer was calibrated using the commercial negative ion calibration mixture from Thermo-Fisher Scientific immediately prior to submission of the batch analysis of 24 extracted samples. Fragmentation data (i.e., MS/MS) for samples was collected in an unsupervised “Top 5” data-dependent manner using 35 eV of collision energy.

### 3.10. Statistical Analyses

Statistical analyses were performed with GraphPad Prism 6 software (GraphPad Software Inc., San Diego, CA, USA).

## 4. Conclusions

In the present study, active propolis compounds from Eastern Canada were extracted with various solvents of different polarities and with three different extraction methods. Despite the differences in the three extraction methods, extracts obtained with polar solvents such as ethanol and methanol had similar TPC and TFC. These same extracts also had the best ability to eliminate free radicals and had inhibitory potential of the biosynthesis of 5-LO products similar to the only product marketed as a 5-LO inhibitor. Acetone extracts obtained with UAE showed the highest antiradical potential (IC_50_ = 48 µg/mL). The effect of ultrasound, even at room temperature, seems to favor the extraction of molecules with high antiradical activity. Extracts obtained following UAE with ethyl acetate, dichloromethane, or ethyl acetate/hexane (1:1) showed the same activity against 5-LO as extracts obtained with much more polar solvents like ethanol and methanol.

A total of twenty-one targeted phenolic acid and flavonoid compounds were detected by mass spectrometry with an additional twenty-five tentative compound assignments based on exact mass measurements. Fragmentation data was collected for all compounds that may prove useful for comparison of future propolis chemical profiling experiments.

The current study is the first of its kind to concurrently investigate solvent polarity as well as extraction techniques of propolis. Ethanol-based Soxhlet or maceration are still the preferred methods of active propolis compounds extraction. But a general societal shift towards more environment-friendly methods has led to the development of greener methods such as UAE. This relatively new method regarding propolis extraction has shown promise in terms of propolis yields, shorter extraction times, reduction in solvent use and biological activity of extracts.

## Figures and Tables

**Table 1:** Extraction yield of propolis with different extraction methods and solvents.

Solvents		m (g/5 g of Dry Propolis)		
		SE	MAE	UAE
Water	Mean	0.047	0.044	0.065
	CI	0.013 to 0.081	0.034 to 0.055	0.033 to 0.096
Methanol	Mean	1.774	1.362	1.352
	CI	1.553 to 1.995	1.245 to 1.478	1.283 to 1.420
Ethanol	Mean	1.936	0.876	0.737
	CI	1.853 to 2.018	0.785 to 0.968	0.535 to 0.938
Acetone	Mean	1.614	0.825	1.466
	CI	1.251 to 1.977	0.680 to 0.971	1.299 to 1.632
EtOAc	Mean	2.283	1.079	0.768
	CI	1.960 to 2.605	0.790 to 1.368	0.329 to 1.207
Dichloromethane	Mean	2.08	0.937	1.028
	CI	1.493 to 2.680	0.619 to 1.255	0.933 to 1.122
EtOAc:Hex (1:1)	Mean	0.761	0.425	0.153
	CI	0.612 to 0.910	0.336 to 0.515	0.144 to 0.162
EtOAc:Hex (4:1)	Mean	0.781	0.641	0.335
	CI	0.555 to 1.007	0.541 to 0.741	0.226 to 0.443
EtOAc:Hex (1:4)	Mean	1.091	0.483	0.31
	CI	0.868 to 1.313	0.258 to 0.709	0.252 to 0.368
Hexane	Mean	0.599	0.324	0.53
	CI	0.275 to 0.923	0.244 to 0.403	0.397 to 0.664

Values are means from 3 independent experiments, CI (confidence interval) = 95%.

**Table 2:** Antioxidant activity of propolis extracts measured by free radicals scavenging.

Solvents		IC_50_ (µg/mL)		
		SE	MAE	UAE
Water	Mean	451.8	359.1	301.4
	CI	432.4 to 472.0	354.9 to 363.4	282.8 to 321.1
Methanol	Mean	76.01	63.22	79.12
	CI	72.13 to 80.10	61.54 to 64.94	74.60 to 83.92
Ethanol	Mean	59.03	70.03	74.38
	CI	57.97 to 60.10	58.90 to 83.26	68.91 to 80.27
Acetone	Mean	124.9	88.54	48.11
	CI	120.8 to 129.2	84.13 to 93.18	44.97 to 51.48
EtOAc	Mean	118.3	120.6	97.24
	CI	114.7 to 122.1	110.4 to 131.8	87.76 to 107.8
Dichloromethane	Mean	231.7	102.3	186.8
	CI	213.6 to 251.4	80.95 to 129.3	174.0 to 200.6
EtOAc:Hex (1:1)	Mean	~1290	246.9	188.2
	CI	(Very wide)	210.2 to 289.9	176.5 to 200.7
EtOAc:Hex (4:1)	Mean	590.6	140.5	187.5
	CI	482.2 to 723.4	131.6 to 149.9	170.1 to 206.5
EtOAc:Hex (1:4)	Mean	378.0	480.8	770.4
	CI	356.0 to 401.4	468.6 to 493.4	731.0 to 812.0
Hexane	Mean	NI	NI	NI
Ascorbic acid	Mean	10.91		
	CI	9.55 to 12.47		
Caffeic acid	Mean	8.80		
	CI	8.16 to 9.48		
Quercetin	Mean	6.68		
	CI	5.68 to 7.84		

Values are means from three independent experiments, each performed in duplicate. CI = 95% confidence interval. NI: no inhibition.

**Table 3:** A list of standard compounds identified in extracts of propolis from Eastern Canada (South-East New Brunswick) by LC-MS/MS: All identifications in this table were confirmed with analytical standards.

				[M − H]^−^ (*m*/*z*)			MS/MS Fragments (*m/z*)	
t_R_ (min)	Compound	CAS No.	Formula	Calculated	Experimental	Mass Accuracy (ppm)	Q-Exactive	LTQ-XL
13.1	*p*-Hydroxybenzaldehyde	123-8-0	C_7_H_6_O_2_	121.0284	121.0278	5.0	n.d.	n.d.
13.1	Caffeic acid	331-39-5	C_9_H_8_O_4_	179.0339	179.0337	1.1	135.0426	135.2
14.7	*p*-Coumaric acid	501-98-4	C_9_H_8_O_3_	163.0390	163.0384	3.7	119.0477	119.2
15.2	Ferulic acid	1135-24-6	C_10_H_10_O_4_	193.0495	193.0489	3.1	134.0348, 178.0246, 149.0582	149.2, 134.2, 178.1
15.9	Cinnamic acid	140-10-3	C_9_H_8_O_2_	147.0441	147.0436	3.4	103.0541	103.2
16.2	Benzoic acid	65-85-0	C_7_H_6_O_2_	121.0284	121.0278	5.0	n.d.	n.d.
17.6	Quercetin	117-39-5	C_15_H_10_O_7_	301.0343	301.0332	3.7	151.0011, 286.0462, 178.9960	179.1, 151.1, 273.2
18.0	Ethyl caffeate	102-37-4	C_11_H_12_O_4_	207.0652	207.0645	3.4	179.0325, 135.0426, 161.0219	179.2, 133.2, 161.2
18.8	Apigenin	520-36-5	C_15_H_10_O_5_	269.0444	269.0437	2.6	n.d.	225.1, 149.1, 218.2
19.0	Pinobanksin	548-82-3	C_15_H_12_O_5_	271.0601	271.0593	3.0	253.0486, 225.0536, 215.0691	253.2, 225.2, 215.2
19.1	Kaempferol	520-18-3	C_15_H_10_O_6_	285.0394	285.0385	3.2	145.0269, 139.0374	255.2, 151.1, 229.2
19.2	Isorhamnetin	480-19-3	C_16_H_12_O_7_	315.0499	315.0487	3.8	300.0253	300,2
19.3	3-Methylkaempferol	1592-70-7	C_16_H_12_O_6_	299.0550	299.0539	3.7	284.0308, 165.9882, 121.0270	284.2
20.2	Rhamnetin	90-19-7	C_16_H_12_O_7_	315.0499	315.0486	4.1	165.0168, 121.0270, 300.0253	165.2, 193.1, 300.1
21.4	Isosakuranetin	480-43-3	C_16_H_14_O_5_	285.0757	285.0748	3.2	164.0100, 243.0658, 270.0533	270.17, 243.17, 164.08
21.6	Caffeic acid phenylethyl ester	104594-70-9	C_17_H_16_O_4_	283.0965	283.0955	3.5	179.0325, 135.0426, 161.0219	179.1, 135.2, 161.2
21.6	Chrysin	480-4-0	C_15_H_10_O_4_	253.0495	253.0486	3.6	121.0270, 209.1525	209.2, 224.2, 181.2
22.0	4’-Methylkaempferol	491-54-3	C_16_H_12_O_6_	299.0550	299.0539	3.7	284.0308	284,2
22.0	7-Methylkaempferol	569-92-6	C_16_H_12_O_6_	299.0550	299.0539	3.7	284.0308, 256.0357, 121.0270	165.2, 271.1, 283.1
22.0	Galangin	548-83-4	C_15_H_10_O_5_	269.0444	269.0437	2.6	n.d.	239.2, 197.2, 227.2
22.4	Cinnamyl caffeate	115610-79-2	C_18_H_16_O_4_	295.0965	295.0952	4.4	178.0246, 137.0218, 134.0347	178.1, 134.2, 251.3

n.d: not detected.

**Table 4:** Empirical formula assignments for an additional 25 compounds of high abundance in propolis extracts.

	[M − H]^−^ (*m*/*z*)			[M −H]^−^ ( *m*/*z*)		MS/MS Fragments (*m*/*z*)
t_R_ (min)	Experimental	Peak Intensity	Predicted Formula	Calculated	Mass Accuracy (ppm)	Q-Exactive
14.1	237.0746	3.07 × 10^7^	C_12_H_14_O_5_	237.0757	4.6	145.0269, 119.0477, 163.0375
14.42	151.0374	9.22 × 10^7^	C_8_H_8_O_3_	151.0390	10.6	136.0140
15.39	295.0801	9.89 × 10^6^	C_14_H_16_O_7_	295.0812	3.7	161.0218, 59.0116, 135.0426
15.7	329.1004	5.3 × 10^7^	C_18_H_18_O_6_	329.1020	4.9	145.0269, 119.0477, 163.0375
15.8	359.1110	1.0 × 10^8^	C_19_H_20_O_7_	359.1125	4.2	145.0270, 163.0375, 119.0478
16.1	389.1213	4.0 × 10^7^	C_20_H_22_O_8_	389.1231	4.6	175.0375, 193.0482, 134.0348
16.3	177.0533	5.1 × 10^7^	C_10_H_10_O_3_	177.0546	7.3	162.0297
16.6	279.0854	8.8 × 10^7^	C_14_H_16_O_6_	279.0863	3.2	145.0269, 59.0116, 119.0477
16.6	317.1004	1.1 × 10^8^	C_17_H_18_O_6_	317.1020	5.0	121.0270, 222.9902, 250.9852
16.7	363.1058	3.9 × 10^7^	C_18_H_20_O_8_	363.1074	4.4	121.0270, 269. 0801, 164.0453
16.8	309.0959	2.5 × 10^7^	C_15_H_18_O_7_	309.0969	3.2	59.0116, 175.0375, 294.0724
16.9	251.0905	1.9 × 10^7^	C_13_H_16_O_5_	251.0914	3.6	161.0583, 145.0270, 133.0634
17.8	399.1057	1.1 × 10^7^	C_21_H_20_O_8_	399.1074	4.3	163.0375, 119.0477, 253.0697
18.1	343.1161	3.2 × 10^7^	C_19_H_20_O_6_	343.1176	4.4	147.0426, 164.0453
18.5	387.1420	4.2× 10^7^	C_21_H_24_O_7_	387.1438	4.6	119.0477, 145.0209, 163.0375
18.8	383.1106	7.2 × 10^7^	C_21_H_20_O_7_	383.1125	5.0	163.0375, 119.0477, 145.0269
18.9	301.0695	2.9 × 10^7^	C_16_H_14_O_6_	301.0707	4.0	165.9882, 109.9984, 194.9911
18.9	413.1212	2.8 × 10^7^	C_22_H_22_O_8_	413.1231	4.6	193.0481, 163.0375, 134.0348
19.7	191.0690	3.5 × 10^7^	C_11_H_12_O_3_	191.0703	6.8	145.0269, 119.0477, 163.0375
19.9	249.0749	3.0 × 10^7^	C_13_H_14_O_5_	249.0758	3.6	145.0269, 131.0477, 121.0269
21.2	485.1417	3.0 × 10^7^	C_25_H_26_O_10_	485.1442	5.2	193.0841, 134.0347, 175.0375
21.6	255.0643	1.2 × 10^7^	C_15_H_12_O_4_	255.0652	3.5	151.0011, 213.0533, 83.0114
22.5	253.0850	6.5 × 10^8^	C_16_H_14_O_3_	253.0859	3.6	121.0270, 145.0269, 162.0297
22.9	283.0954	6.0 × 10^7^	C_17_H_16_O_4_	283.0965	3.9	177.0168, 133.0269, 268.0721
23.1	267.1007	4.3 × 10^7^	C_17_H_16_O_3_	267.1016	3.4	119.0477, 163.0375, 145.0269

## Supplementary Materials

The following are available online. Figure S1: Cytotoxicity assay of tested extracts on HEK293 cells. Figure S2: Comparison of LC-MS signals for 45 compounds using SE, UAE and MAE extraction techniques with methanol as the solvent. Figure S3: Comparison of LC-MS signals for 45 compounds using SE, UAE and MAE extraction techniques with ethanol as the solvent. The x- and y-axes have been set to the same value for a quick visual evaluation of the more sensitive method, i.e., negative or positive deviations of the slope from unity. Figure S4: Comparison of LC-MS signals for 45 compounds using SE, UAE and MAE extraction techniques with ethyl acetate as the solvent. Figure S5: Comparison of LC-MS signals for 45 compounds using SE, UAE and MAE extraction techniques with dichloromethane as the solvent. Figure S6: Comparison of LC-MS signals for 45 compounds using SE, UAE and MAE extraction techniques with a 1:4 mixture of hexane and ethyl acetate as the solvent. Figure S7: Comparison of LC-MS signals for 45 compounds using SE, UAE and MAE extraction techniques with a 1:1 mixture of hexane and ethyl acetate as the solvent. Figure S8: Comparison of LC-MS signals for 45 compounds using SE, UAE and MAE extraction techniques with a 4:1 mixture of hexane and ethyl acetate as the solvent. Table S1: The slope and R^2^ values for plots of ion signal intensities in different solvents by the microwave-assisted extraction technique. Table S2: The slope and R^2^ values for plots of ion signal intensities in different solvents by the Soxhlet extraction technique. Table S3. The slope and R^2^ values for plots of ion signal intensities in different solvents by the ultrasound-assisted extraction technique.

## References

[1] Ahn M.R., Kumazawa S., Hamasaka T., Bang K.S., Nakayama T. (2004). Antioxidant activity and constituents of propolis collected in various regions of Korea. J. Agric. Food Chem..

[2] Trusheva B., Popova M., Bankova V., Somova S., Marcucci M.C., Miorin P.L., Pasin F.D.R., Tsvetkova I. (2006). Bioactive constituents of Brazilian red propolis. Evid. Based Complementary Altern. Med..

[3] Silici S., Kutluca S. (2005). Chemical composition and antibacterial activity of propolis collected by three different races of honeybees in the same region. J. Ethnopharmacol..

[4] Huang S., Zhang C.P., Wang K., Li G.Q., Hu F.L. (2014). Recent advances in the chemical composition of propolis. Molecules.

[5] Wilson-Rich N. (2011). Genetic, Individual, and Group Facilitation of Disease Resistance in Honeybees (Apis mellifera) and Two Species of Paper Wasps (P. dominolus and P. fuscatus).

[6] Bankova V. (2005). Chemical diversity of propolis and the problem of standardization. J. Ethnopharmacol..

[7] Celińska-Janowicz K., Zaręba I., Lazarek U., Teul J., Tomczyk M., Pałka J., Miltyk W. (2018). Constituents of propolis: Chrysin, caffeic acid, p-coumarin and ferulic acid iinduce PRODH/POX-dependent apoptosis in human tongue squamous cell carcinoma cell (CAL-27). Front. Pharm..

[8] Khodabakhshi D., Eskandarinia A., Kefayat A., Rafienia M., Navid S., Karbasi S., Moshtaghian J. (2019). In vitro and in vivo performance of a propolis-coated polyurethane wound dressing with high porosity and antibacterial efficacy. Colloids Surf B Biointerfaces.

[9] Thamnopoulos I.I., Michailidis G.F., Fletouris D.J., Badeka A., Kontominas M.G., Angelidis A.S. (2018). Inhibitory activity of propolis against *Listeria monocytogenes* in milk stored under refrigeration. Food Microbiol..

[10] Yildirim A., Duran G.G., Duran N., Jenedi K., Bolgul B.S., Miraloglu M., Muz M. (2016). Antiviral activity of Hatay propolis against replication of herpes simplex virus type 1 and 2. Med. Sci. Monit..

[11] Yangi B., Cengiz Ustuner M., Dincer M., Ozbayer C., Tekin N., Ustuner D., Colak E., Kolac U.K., Entok E. (2018). Propolis protects endotoxin induced acute lung and liver inflammation through attenuating inflammatory responses and oxidative stress. J. Med. Food.

[12] Khurshid Z., Naseem M., Zafar M.S., Najeeb S., Zohaib S. (2017). Propolis: A natural biomaterial for dental and oral health care. J. Dent. Res. Dent. Clin. Dent. Prospect..

[13] Henshaw F.R., Bolton T., Nube V., Hood A., Veldhoen D., Pfrunder L., McKew G.L., Macleod C., McLennan S.V., Twigg S.M. (2014). Topical application of the beehive protectant propolis is well tolerated and improves human diabetic foot ulcer healing in a prospective feasibility study. J. Diabetes Its Complicat..

[14] Rzepecka-Stojko A., Stojko J., Kurek-Gorecka A., Gorecki M., Kabala-Dzok A., Kubina R., Mozdzierz A., Buszman E. (2015). Polyphenols from bee pollen: Structure, absorption, metabolism and biological activity. Molecules.

[15] Bonamigo T., Campos J.F., Oliveira A.S., Torquato H.F.V., Balestieri J.B.P., Cardoso C.A.L., Paredes-Gamero E.J., de Picoli Souza K., Dos Santos E.L. (2017). Antioxidant and cytotoxic activity of propolis of Plebeia droryana and Apis mellifera (Hymenoptera, Apidae) from the brazilian Cerrado biome. PLoS ONE.

[16] Werz O. (2007). 5-lipoxygenase: Cellular biology and molecular pharmacology. Curr. Drug Targets-Inflamm. Allergy.

[17] Peters-Golden M., Henderson Jr W.R. (2007). Leukotrienes. N. Engl. J. Med..

[18] Boudreau L.H., Maillet J., LeBlanc L.M., Jean-Francois J., Touaibia M., Flamand N., Surette M.E. (2012). Caffeic acid phenethyl ester and its amide analogue are potent inhibitors of leukotriene biosynthesis in human polymorphonuclear leukocytes. PLoS ONE.

[19] Bittencourt M.L.F., Ribeiro P.R., Franco R.L.P., Hilhorst H.W.M., de Castro R.D., Fernandez L.G. (2015). Metabolite profiling, antioxidant and antibacterial activities of Brazilian propolis: Use of correlation and multivariate analyses to identify potential bioactive compounds. Food Res. Int..

[20] Gomez-Caravaca A.M., Gomez-Romero M., Arraez-Roman D., Segura-Carretero A., Fernandez-Gutierrez A. (2006). Advances in the analysis of phenolic compounds in products derived from bees. J. Pharm. Biomed. Anal..

[21] Viuda-Martos M., Ruiz-Navajas Y., Fernandez-Lopez J., Perez-Alvarez J.A. (2008). Functional properties of honey, propolis and royal jelly. J. Food Sci..

[22] Cao J., Peng L.Q., Du L.J., Zhang Q.D., Xu J.J. (2017). Ultrasound –assisted ionic liquid-based micellar extraction combined with microcrystalline cellulose as sorbent in dispersive microextraction for the determination of phenolic compounds in propolis. Anal. Chim. Acta.

[23] Darendeliogliu E., Aykutoglu G., Tartik M., Baydas G. (2016). Turkish propolis protects human endothelial cells in vitro from homocysteine-induced apoptosis. Acta Histochem..

[24] Machado B.A.S., De Abreu Barreto G., Costa A.S., Costa S.S., Silva R.P.D., Da Silva D.F. (2015). Determination of parameters for the supercritical extraction of antioxidant compounds from green propolis using carbon dioxide and ethanol as co-solvent. PLoS ONE.

[25] Trusheva B., Trunkova D., Bankova V. (2007). Different extraction methods of biologically active components from propolis: A preliminary study. Chem. Cent. J..

[26] Reis J.H.dO., Barreto G.dA., Cerquiera J.C., Anjos J.P.D., Andrade L.N., Padilha F.F., Machado B.A.S. (2019). Evaluation of the antioxidant profile and cytotoxic activity of red propolis extracts from different regions of northeastern Brazil obtained by conventional and ultrasound-assisted extraction. PLoS ONE.

[27] Scepankova H., Martins M., Estevinho L., Delgadillo I., Saraiva J.A. (2018). Enhancement of bioactivity of natural extracts by non-thermal high hydrostatic pressure extraction. Plant Foods Hum. Nutr..

[28] Devequi-Nunes D., Machado B.A.S., Barreto G.dA., Reboucas Silva J., da Silva D.F., da Rocha J.L.C. (2018). Chemical characterization and biological activity of six different extracts of propolis through conventional methods and supercritical extraction. PLoS ONE.

[29] Andrade J.K.S., Denaddai M., Andrade G.R.S., da Cuhna Nascimento C., Barbosa P.F., Jesus M.S. (2018). Development and characterization of microcapsules containing spray dried powder obtained from Brazilian brown, green and red propolis. Food Res. Int..

[30] Sadhana N., Lohidasan S., Mahadik K.R. (2017). Marker-based standardization and investigation of nutraceutical potential of Indian propolis. J. Integr. Med..

[31] Radmark O., Werz O., Steinhilber D., Samuelsson B. (2007). 5-Lipoxygenase: Regulation of expression and enzyme activity. Trends Biochem. Sci..

[32] Doiron J., Boudreau L.H., Picot N., Villebonet B., Surette M.E., Touaibia M. (2009). Synthesis and 5-Lipoxygenase Inhibitory Activity of New Cinnamoyl and Caffeoylclusters. Bioorg. Med. Chem. Lett..

[33] Sawaya A.C.H.F., Tomazela D.M., Cunha I.B.S., Bankova V.S., Marcucci M.C., Custodio A.R., Eberlin M.N. (2004). Electrospray ionization mass spectrometry fingerprinting of propolis. Analyst.

[34] Trudic B., Andelkovic B., Orlovic S., Tesevic V., Pilipovic A., Cvetkovic M., Stankovic J. (2016). HPLC/MS—TOF analysis of surface resins from three poplar clones grown in Serbia. South-East Eur. For..

[35] Costa A.G., Yoshida N.C., Garcez W.S., Perdomo R.T., Matos M.C., Garcez F.R. (2020). Metabolomics approach expands the classification of propolis samples from midwest Brazil. J. Nat. Prod..

[36] Shi H., Yang H., Zhang X., Yu L. (2012). Identification and quantification of phytochemical composition and anti-inflammatory and radical scavenging properties of methanolic extracts of Chinese propolis. J. Agric. Food Chem..

[37] Yuan Y., Zheng S., Zeng L., Deng Z., Zhang B., Li H. (2019). The phenolic compounds, metabolites, and antioxidant activity of propolis extracted by ultrasound-assisted method. J. Food Sci..

[38] Greenaway W., May J., Scaysbrook T., Whatley F.R. (1991). Identification by gas chromatography-mass spectrometry of 150 compounds in propolis. Z. Nat. C.

[39] Romero M., Freire J., Pastene E., Garcia A., Aranda M., Gonzalez C. (2019). Propolis polyphenolic compounds affect the viability and structure of *Heliicobacter pyroli in vitro*. Braz. J. Pharmacogn..

[40] Zingue S., Nde C.B.M., Michel T., Ndinteh D.T., Tchatchou J., Adamou M., Fernandez X., Fohouo F.N.T., Clyne C., Njamen D. (2017). Ethanol-extracted Cameroonian propolis exerts estrogenic effects and alleviates hot flushes in ovariectomized Wistar rats. BMC Complementary Altern. Med..

[41] deGroot A.C., Popova M.P., Bankova V.S. (2014). An update on the constituents of poplar-type propolis.

[42] Bakdash A., Almohammadi O.H., Taha N.A., Abu-Rumman A., Kumar S. (2018). Chemical composition of propolis from the Baha region in Saudi Arabia. Czech J. Food Sci..

[43] Allain E.P., Boudreau L.H., Flamand N., Surette M.E. (2015). The Intracellular Localisation and Phosphorylation Profile of the Human 5-Lipoxygenase Δ13 Isoform Differs from That of Its Full Length Counterpart. PLoS ONE.

