# Supplementary material for: Extraction, Antioxidant Capacity, 5-Lipoxygenase Inhibition, and Phytochemical Composition of Propolis from Eastern Canada
Source: Molecules. 2020 May 21;25(10):2397. doi: 10.3390/molecules25102397 (PMC7287732; doi:10.3390/molecules25102397)
Supplement: Supplementary file 1 [file molecules-25-02397-s001.pdf]

# Extraction, Antioxidant Capacity, 5-Lipoxygenase Inhibition, and Phytochemical Composition of Propolis from Eastern Canada

**Mariama Sambou [1], Jacques Jean-François [2], Fanta J. Ndongou Moutombi [1], Jérémie A. Doiron [1], Mathieu P.A. Hébert [1], Andrew P. Joy [3], Ngoc-Nu Mai-Thi [3], David A. Barnett [3], Marc E. Surette [1], Luc H. Boudreau [1] and Mohamed Touaibia [1,*]**

[1] Department of Chemistry and Biochemistry, Université de Moncton, Moncton, NB E1A 3E9, Canada.
[2] Department of Chemistry, Texas Lutheran University, Seguin, TX 78155, USA.
[3] Atlantic Cancer Research Institute, Moncton, NB E1C 8X3, Canada
* Correspondence: mohamed.touaibia@umoncton.ca

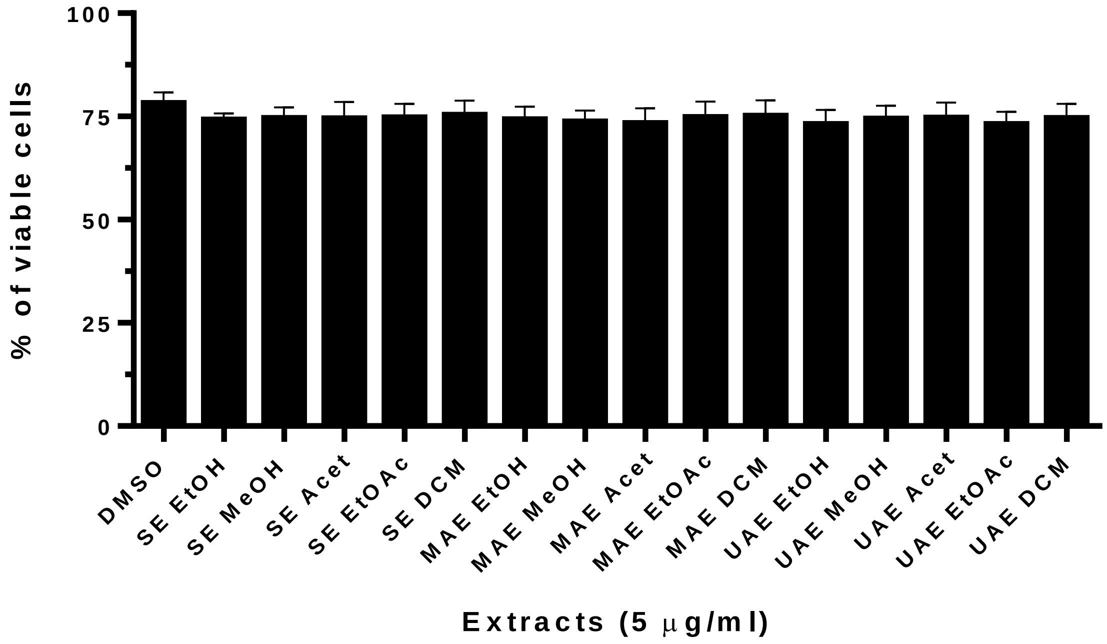


**Figure S1:** Cytotoxicity assay of tested extracts on HEK293 cells. HEK293 were detached with Accutase (Corning), resuspended at 5 x $10^5$ cells/ml in PBS and then incubated in the presence of the indicated compounds at 5 μg/ml or vehicle (DMSO, 0.1%) for 20 minutes. Cell viability was immediately assessed by flow cytometry (Attune NxT, ThermoFisher) following staining with annexin V Alexa Fluor 647 and zombie aqua™ (Biolegend). No statistically differences, from DMSO control, were observed following one-way analysis of variance followed by Dunnett's multiple comparisons test.

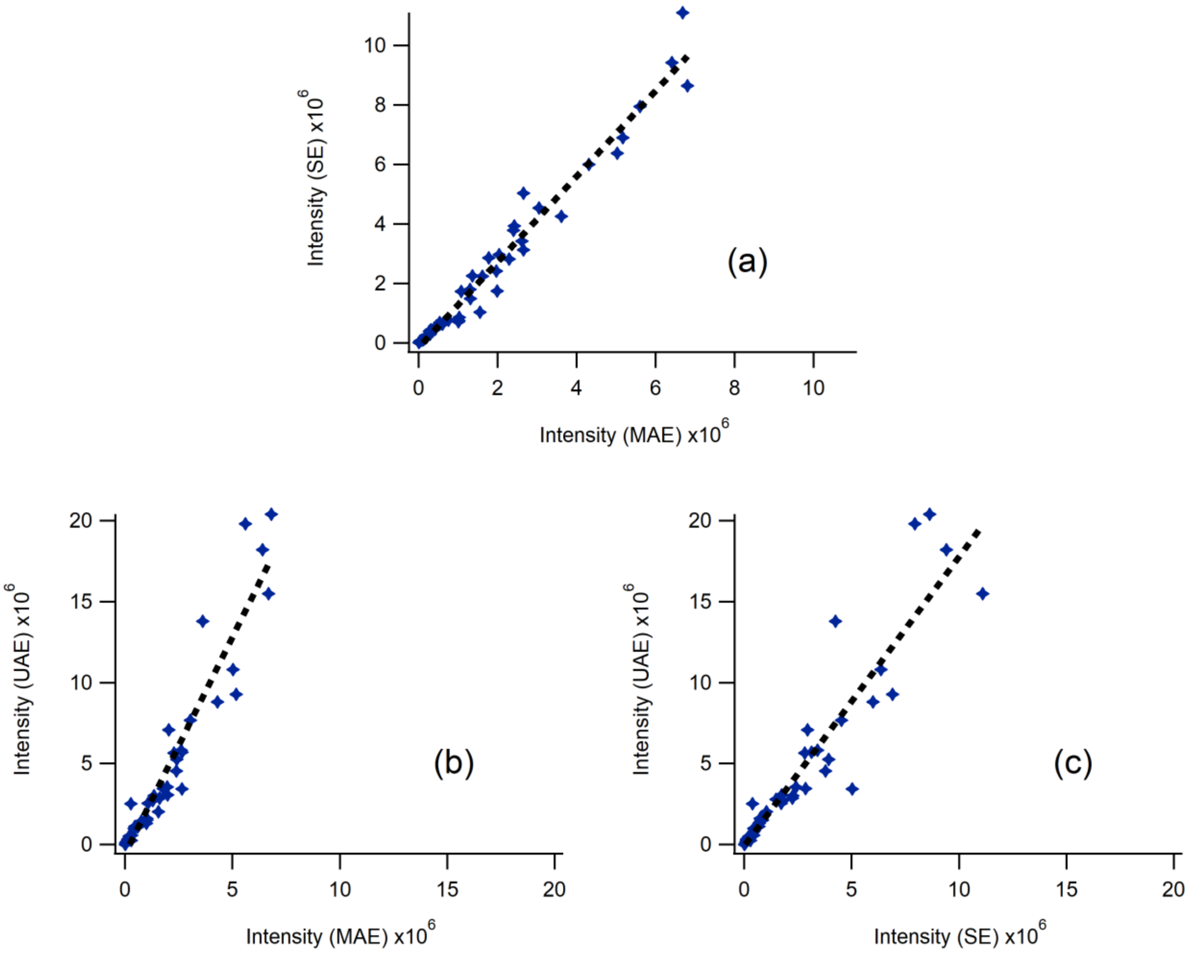


**Figure S2**. Comparison of LC-MS signals for 45 compounds using SE, UAE and MAE extraction techniques with methanol as the solvent. Note that the UAE data was recollected several days later than the SE and MAE data due to an unfortunate loss of the original data. The UAE numbers appear to be marginally higher than expected which could be due to a batch effect following routine maintenance of the mass spectrometer. The x- and y-axes have been set to the same value for a quick visual evaluation of the more sensitive method, i.e., negative or positive deviations of the slope from unity.

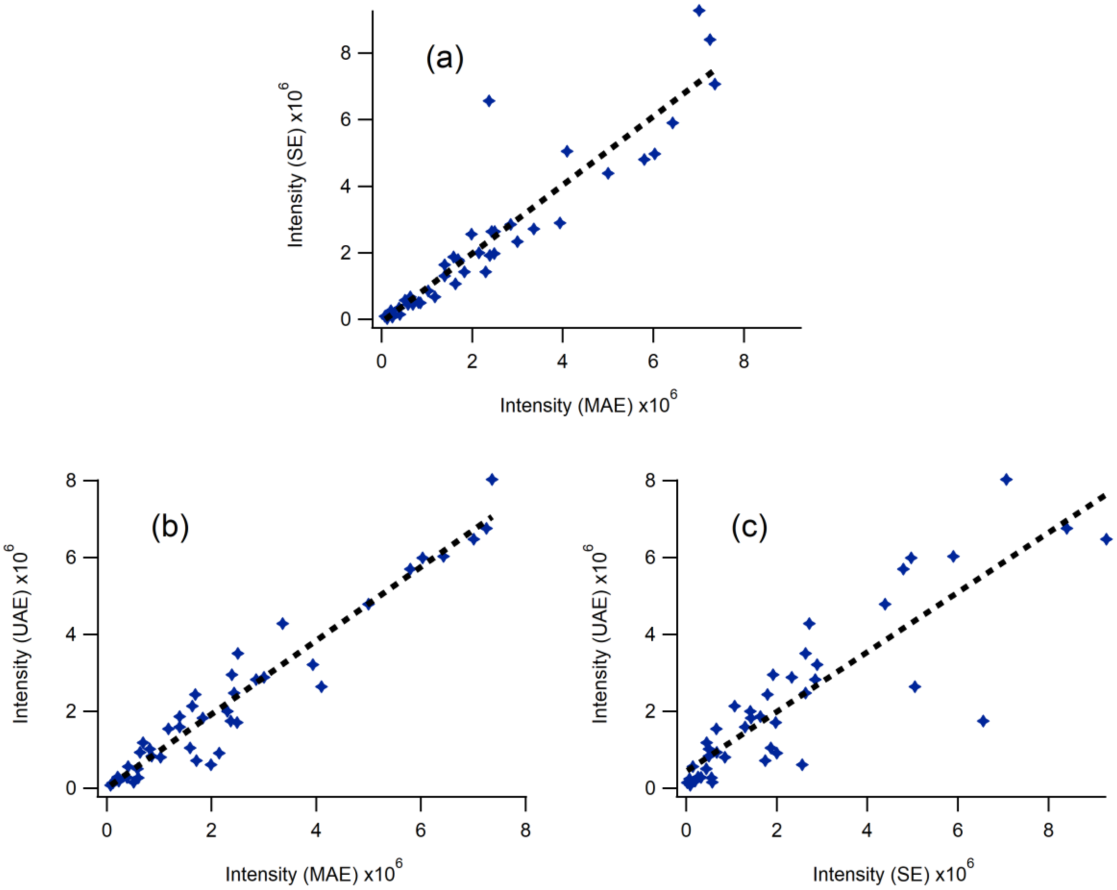


**Figure S3**. Comparison of LC-MS signals for 45 compounds using SE, UAE and MAE extraction techniques with ethanol as the solvent. The x- and y-axes have been set to the same value for a quick visual evaluation of the more sensitive method, i.e., negative or positive deviations of the slope from unity.

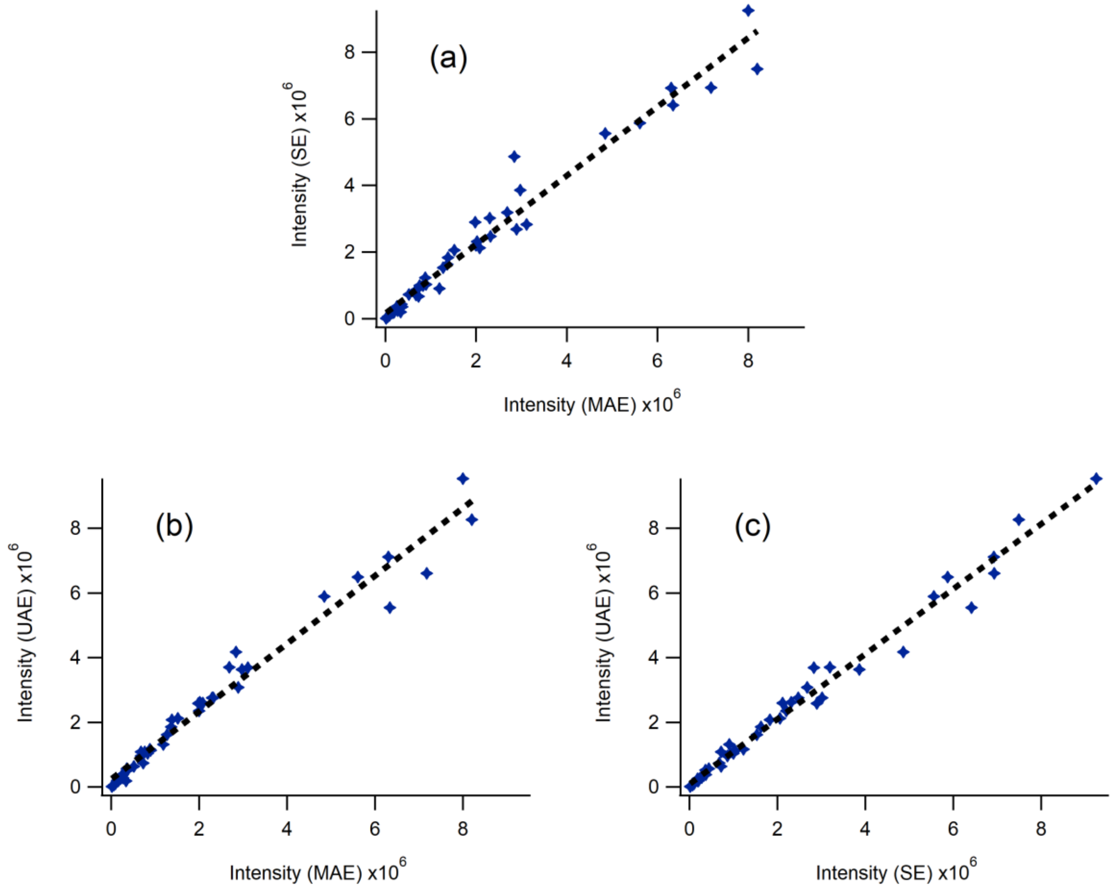


**Figure S4**. Comparison of LC-MS signals for 45 compounds using SE, UAE and MAE extraction techniques with ethyl acetate as the solvent. The x- and y-axes have been set to the same value for a quick visual evaluation of the more sensitive method, i.e., negative or positive deviations of the slope from unity.

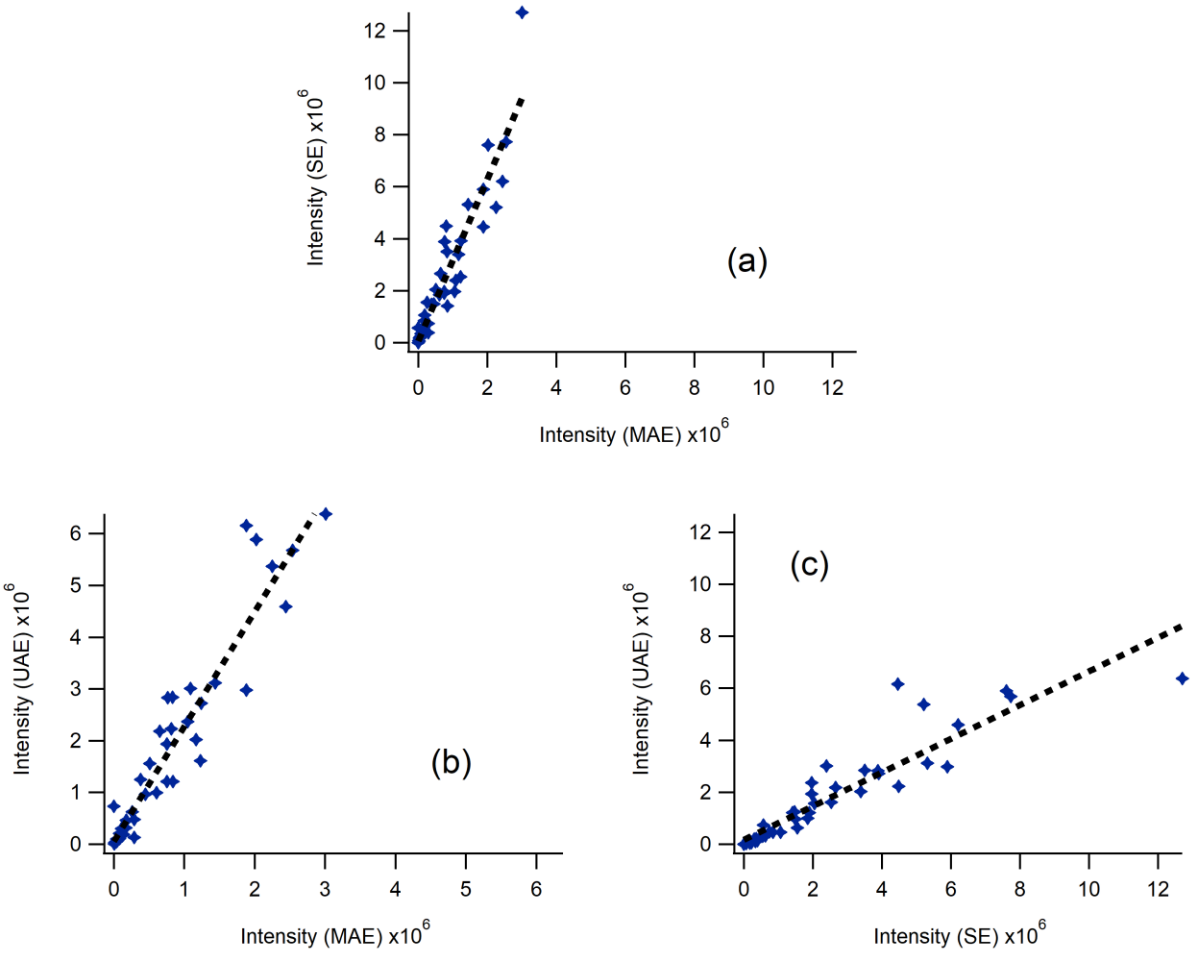


**Figure S5**. Comparison of LC-MS signals for 45 compounds using SE, UAE and MAE extraction techniques with dichloromethane as the solvent. The x- and y-axes have been set to the same value for a quick visual evaluation of the more sensitive method, i.e., negative or positive deviations of the slope from unity.

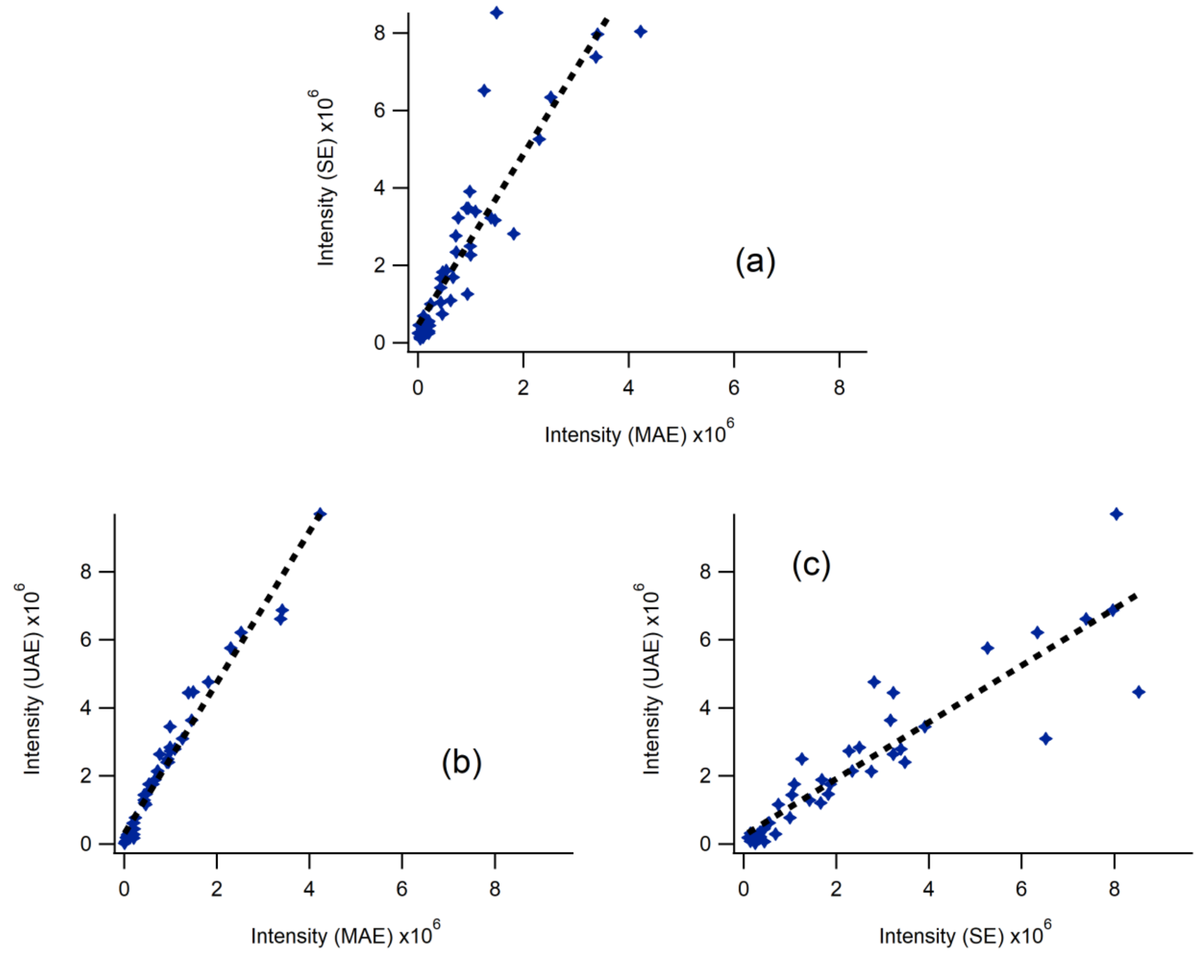


**Figure S6**. Comparison of LC-MS signals for 45 compounds using SE, UAE and MAE extraction techniques with a 1:4 mixture of hexane and ethyl acetate as the solvent. The x- and y-axes have been set to the same value for a quick visual evaluation of the more sensitive method, i.e., negative or positive deviations of the slope from unity.

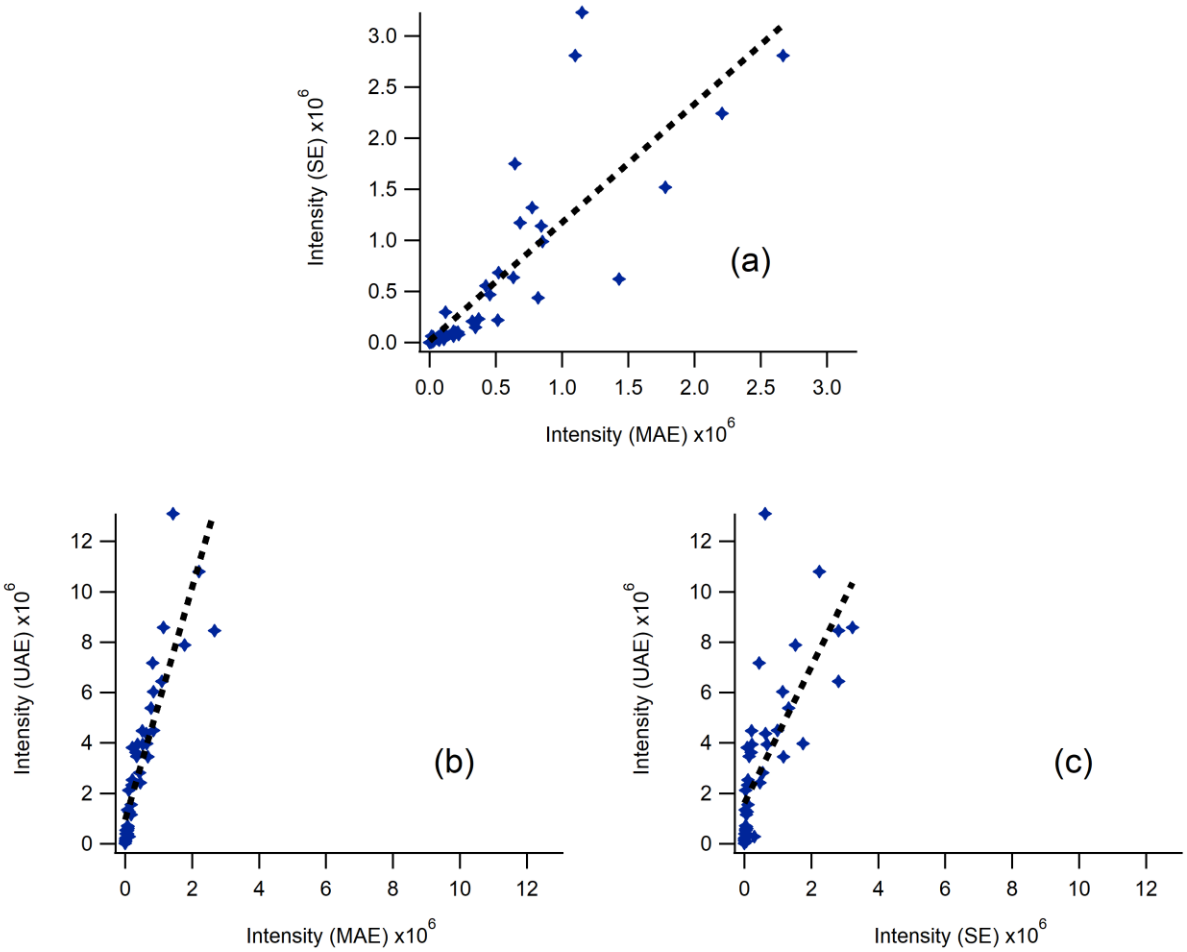


**Figure S7**. Comparison of LC-MS signals for 45 compounds using SE, UAE and MAE extraction techniques with a 1:1 mixture of hexane and ethyl acetate as the solvent. The x- and y-axes have been set to the same value for a quick visual evaluation of the more sensitive method, i.e., negative or positive deviations of the slope from unity.

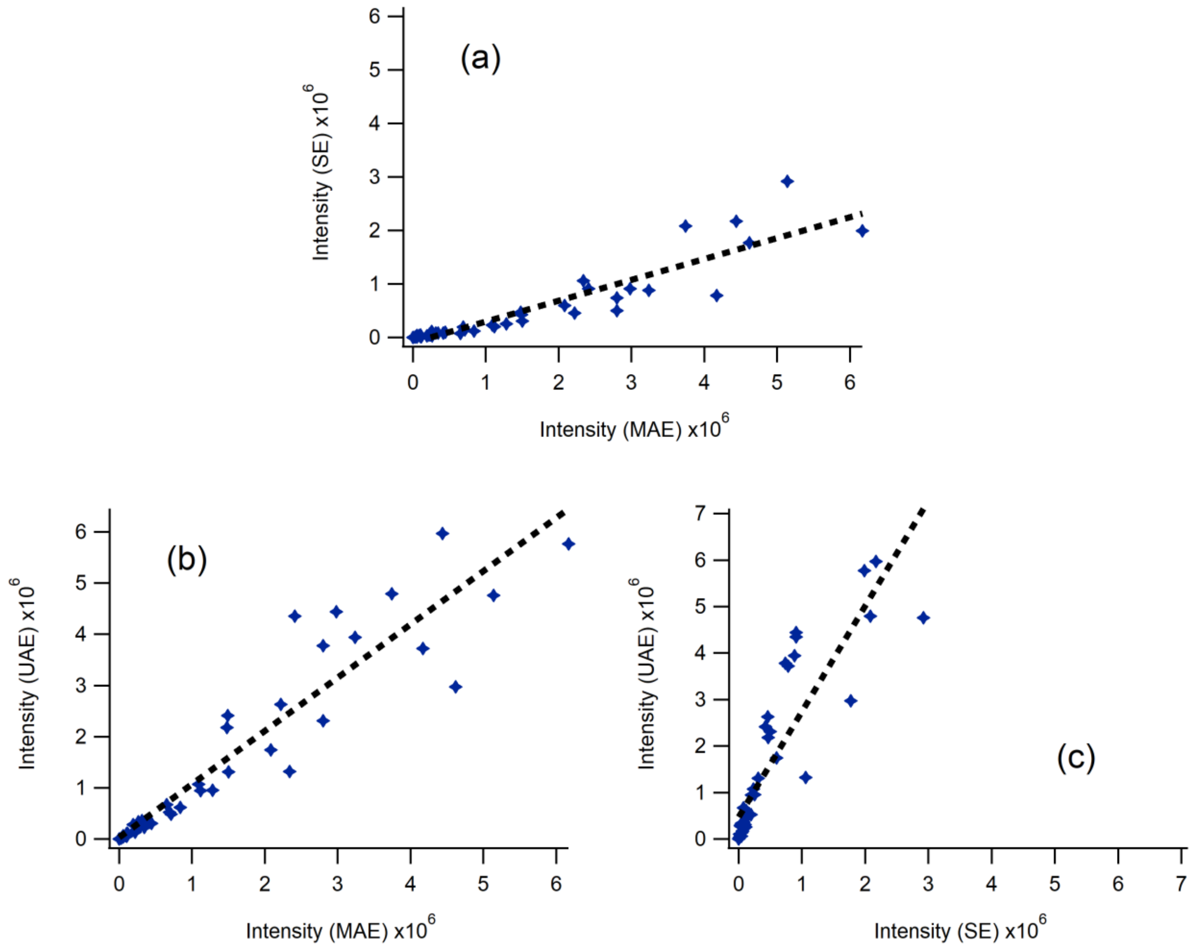


**Figure S8**. Comparison of LC-MS signals for 45 compounds using SE, UAE and MAE extraction techniques with a 4:1 mixture of hexane and ethyl acetate as the solvent. The x- and y-axes have been set to the same value for a quick visual evaluation of the more sensitive method, i.e., negative or positive deviations of the slope from unity.

**Table S1**. The slope and $R^2$ values for plots of ion signal intensities in different solvents by the microwave-assisted extraction technique. Solvents in the columns are plotted on the ordinate axis while rows are plotted on the abscissa.

| Solvent | MeOH | EtOH | Acetone | EtOAc | DCM | 1:4 Hex:EtOAc | 1:1 Hex:EtOAc | 4:1 Hex:EtOAc |
|---|---|---|---|---|---|---|---|---|
| **MeOH** | | 1.05 (0.932) | 1.22 (0.956) | 1.15 (0.954) | 0.34 (0.663) | 0.48 (0.897) | 0.24 (0.604) | 0.62 (0.533) |
| **EtOH** | | | 1.08 (0.888) | 1.0 (0.901) | 0.28 (0.560) | 0.42 (0.821) | 0.21 (0.498) | 0.50 (0.423) |
| **Acetone** | | | | 0.93 (0.988) | 0.29 (0.743) | 0.40 (0.966) | 0.20 (0.631) | 0.51 (0.567) |
| **EtOAc** | | | | | 0.30 (0.698) | 0.42 (0.937) | 0.21 (0.599) | 0.52 (0.528) |
| **DCM** | | | | | | 1.12 (0.838) | 0.67 (0.789) | 1.87 (0.843) |
| **1:4 Hex:EtOAc** | | | | | | | 0.52 (0.727) | 1.35 (0.660) |
| **1:1 Hex:EtOAc** | | | | | | | | 2.58 (0.918) |
| **4:1 Hex:EtOAc** | | | | | | | | |

**Table S2**. The slope and $R^2$ values for plots of ion signal intensities in different solvents by the Soxhlet extraction technique. Solvents in the columns are plotted on the ordinate axis while rows are plotted on the abscissa.

| Solvent | MeOH | EtOH | Acetone | EtOAc | DCM | 1:4 Hex:EtOAc | 1:1 Hex:EtOAc | 4:1 Hex:EtOAc |
|---|---|---|---|---|---|---|---|---|
| **MeOH** | | 0.71 (0.738) | 0.79 (0.951) | 0.82 (0.952) | 0.83 (0.787) | 0.75 (0.752) | 0.15 (0.259) | 0.15 (0.394) |
| **EtOH** | | | 0.82 (0.699) | 0.83 (0.660) | 0.79 (0.488) | 0.74 (0.506) | 0.16 (0.190) | 0.17 (0.328) |
| **Acetone** | | | | 1.04 (0.983) | 1.08 (0.865) | 0.92 (0.741) | 0.18 (0.243) | 0.19 (0.379) |
| **EtOAc** | | | | | 1.03 (0.860) | 0.89 (0.766) | 0.18 (0.253) | 0.18 (0.382) |
| **DCM** | | | | | | 0.82 (0.788) | 0.17 (0.284) | 0.16 (0.376) |
| **1:4 Hex:EtOAc** | | | | | | | 0.29 (0.682) | 0.25 (0.757) |
| **1:1 Hex:EtOAc** | | | | | | | | 0.80 (0.938) |
| **4:1 Hex:EtOAc** | | | | | | | | |

**Table S3**. The slope and $R^2$ values for plots of ion signal intensities in different solvents by the ultrasound-assisted extraction technique. Solvents in the columns are plotted on the ordinate axis while rows are plotted on the abscissa.

| **Solvent** | **MeOH** | **EtOH** | **Acetone** | **EtOAc** | **DCM** | **1:4 Hex:EtOAc** | **1:1 Hex:EtOAc** | **4:1 Hex:EtOAc** |
|---|---|---|---|---|---|---|---|---|
| **MeOH** | | 0.35 (0.820) | 0.45 (0.797) | 0.39 (0.749) | 0.27 (0.623) | 0.36 (0.773) | 0.44 (0.568) | 0.15 (0.197) |
| **EtOH** | | | 1.29 (0.982) | 1.14 (0.968) | 0.81 (0.821) | 1.04 (0.952) | 1.30 (0.751) | 0.46 (0.286) |
| **Acetone** | | | | 0.88 (0.986) | 0.63 (0.839) | 0.81 (0.970) | 1.02 (0.778) | 0.37 (0.304) |
| **EtOAc** | | | | | 0.69 (0.797) | 0.90 (0.945) | 1.12 (0.743) | 0.39 (0.273) |
| **DCM** | | | | | | 1.12 (0.880) | 1.56 (0.866) | 0.67 (0.479) |
| **1:4 Hex:EtOAc** | | | | | | | 1.32 (0.878) | 0.52 (0.406) |
| **1:1 Hex:EtOAc** | | | | | | | | 0.49 (0.706) |
| **4:1 Hex:EtOAc** | | | | | | | | |
